# Streamlining of Simple Sequence Repeat Data Mining Methodologies and Pipelines for Crop Scanning

**DOI:** 10.3390/plants13182619

**Published:** 2024-09-19

**Authors:** Subramaniam Geethanjali, Palchamy Kadirvel, Mahender Anumalla, Nithyananth Hemanth Sadhana, Anandan Annamalai, Jauhar Ali

**Affiliations:** 1 Department of Plant Biotechnology, Centre for Plant Molecular Biology and Biotechnology, Tamil Nadu Agricultural University, Coimbatore 641003, India; geethanjalitnau@yahoo.com (S.G.); sadhanariya17@gmail.com (N.H.S.); 2 Crop Improvement Section, ICAR-Indian Institute of Oilseeds Research, Rajendranagar, Hyderabad 500030, India; kadirvel.palchamy@gmail.com; 3 Rice Breeding Innovation Platform, International Rice Research Institute (IRRI), Los Baños 4031, Laguna, Philippines; m.anumalla@irri.org; 4 IRRI South Asia Hub, Patancheru, Hyderabad 502324, India; 5 Indian Council of Agricultural Research (ICAR), Indian Institute of Seed Science, Bengaluru 560065, India

**Keywords:** molecular markers, simple sequence repeats, microsatellites, SSR data mining, primer designing, genomic resources, microsatellite databases

## Abstract

Genetic markers are powerful tools for understanding genetic diversity and the molecular basis of traits, ushering in a new era of molecular breeding in crops. Over the past 50 years, DNA markers have rapidly changed, moving from hybridization-based and second-generation-based to sequence-based markers. Simple sequence repeats (SSRs) are the ideal markers in plant breeding, and they have numerous desirable properties, including their repeatability, codominance, multi-allelic nature, and locus specificity. They can be generated from any species, which requires prior sequence knowledge. SSRs may serve as evolutionary tuning knobs, allowing for rapid identification and adaptation to new circumstances. The evaluations published thus far have mostly ignored SSR polymorphism and gene evolution due to a lack of data regarding the precise placements of SSRs on chromosomes. However, NGS technologies have made it possible to produce high-throughput SSRs for any species using massive volumes of genomic sequence data that can be generated fast and at a minimal cost. Though SNP markers are gradually replacing the erstwhile DNA marker systems, SSRs remain the markers of choice in orphan crops due to the lack of genomic resources at the reference level and their adaptability to resource-limited labor. Several bioinformatic approaches and tools have evolved to handle genomic sequences to identify SSRs and generate primers for genotyping applications in plant breeding projects. This paper includes the currently available methodologies for producing SSR markers, genomic resource databases, and computational tools/pipelines for SSR data mining and primer generation. This review aims to provide a ‘one-stop shop’ of information to help each new user carefully select tools for identifying and utilizing SSRs in genetic research and breeding programs.

## 1. Introduction

The genome sequencing era has provided better insights into the DNA sequence organization of several organisms, from primitive prokaryotes to highly evolved eukaryotes. While only a small fraction (4–5%) of the genome constitutes genes and functional elements, more than half of the genome had unknown functions and was referred to earlier as junk DNA or dark matter of the genome. However, it has been established that this junk matter is a factory of highly essential regulatory tuning knobs with biochemical functions associated with gene regulation and expression [1]. Noncoding and repetitive elements constituting the heterochromatic region have been identified as characteristic features of this “dark matter” of the genome. During the evolutionary process, these elements accumulated, resulting in a vast expansion in the genome size and complexity of organisms. More than half of the mammalian genome, specifically two-thirds of the human genome, comprises repetitive elements [2,3]. 

Repetitive elements are sequence motifs repeated more than once in the genome. They are categorized into two classes, viz., dispersed repeats and tandem repeats. Dispersed repeats are repeat motifs randomly scattered throughout the genome. These repeats comprise mobile elements like DNA transposons and retrotransposons. Tandem repeats are sequence motifs in iterated copies occurring consecutively along a DNA strand. This class of repeats constitutes the satellite DNA, minisatellites, and microsatellites [4]. Among repetitive elements, tandem repeats are particularly interesting to biologists as they are vital in the evolutionary process and are robust genetic markers for molecular studies [5,6,7,8,9]. Importantly, they are beneficial for measuring genetic distance and capturing diversity, estimating gene flow and the crossing-over rate, and generating integrated maps for full-sib families for linkage and association mapping studies [10]. However, in crops lacking genome sequence information, library construction, which is time-consuming and tedious, was the only viable method for extracting repeat sequences. On the contrary, in silico data mining methodologies provide a rapid and cost-effective approach to developing SSR markers. A vast quantity of sequence data from multiple sequencing initiatives is a valuable resource for SSR data mining. To identify repeats, these data must be processed and computationally analyzed; subsequently, the repeats must be characterized, and primers must be designed to utilize them as genetic markers. Several computational tools and pipelines have been developed for SSR data mining; nonetheless, inconsistencies emerge in the output of different tools, rendering no single tool an ideal selection for identifying and characterizing every type of repeat. Therefore, the selection of computational tools is contingent upon the user’s specifications, the algorithms implemented, computing speed, and adaptability. 

To provide a novice user with a streamlined understanding of data mining methodologies, “one-stop shop” pipelines that integrate various software tools for preprocessing, repeat identification, primer designing with default settings, and data visualization would constitute a straightforward alternative. In this review, we describe the development and applications for designing SSR markers using various web tools and algorithms that can be user-friendly in genetic research and breeding programs, which we believe could be useful for future crop improvement programs. 

## 2. SSRs: A Robust Framework for Crop Genetic Markers

Due to their abundance, genome-wide distribution, locus specificity, codominance, and poly-allelic nature, SSRs have recently emerged as the molecular markers of choice for crop improvement projects among breeders. SSRs are prevalent in plant genomes at a frequency of about 64 kilobases (kb) [11]. While developing SSR markers is a costly and time-consuming ordeal, the benefits of these markers in areas such as marker-assisted selection, genetic mapping, QTL mapping, DNA fingerprinting, and germplasm characterization are substantial [6,11]. Various types of nucleotide repeats are found in the genome; these include mono-, di-, tri-, tetra-, penta-, and hexanucleotide repeats. Depending upon the repetitive architecture of the motifs, they are classified as perfect, imperfect, or compound repeats. Because of polymerase slippage during DNA replication, these regions of the genome accumulate mutations at a faster rate than others [6]. They are also flanked by conserved sequences, making it possible to design PCR primers that amplify a specific locus.

### 2.1. Development of SSR Markers

#### 2.1.1. Genomic Library Construction

The traditional method for extracting repeat-containing DNA fragments involves building small insert genomic libraries, which entails fragmenting genomic DNA, ligating it into a plasmid vector, and then transforming the resulting bacterial cells. The next step is to use repetitive sequences as probes to screen the bacterial colonies. Candidate clones hybridizing to the probes are extracted and sequenced for primer development. Several protocols for generating SSR-enriched libraries using selective hybridization and primer extension enrichment strategies have been developed, varying in their efficiency [12]. Techen et al. developed a simplified protocol that works well across species by testing various adapter and ligation methods and optimizing several parameters [13]. Building such a genomic library is arduous, time-consuming, and expensive. Despite these limitations, it was the sole essential approach for developing microsatellite markers in crops lacking genome sequence information, especially in the pre-genomics era. Nevertheless, the reliance on genomic library construction for developing SSR markers has been diminished due to revolutionary second- and third-generation sequencing technologies.

#### 2.1.2. In Silico Approaches

With the sequencing of the first eukaryotic genome (yeast, *Saccharomyces cerevisiae*) in 1996, in silico approaches based on bioinformatic tools opened up new avenues for developing SSR markers. The development of several high-throughput sequencing platforms at a reasonable cost helped to generate enormous genomic data that serve as an alternative repository to search for repeat motifs, thereby avoiding laborious library construction protocols. As access to public databases comes free of cost, developing SSR markers from these databases is an easier, more economical, and faster approach. The data mining approach for SSR marker development is being used extensively in several species. To perform in silico data mining, the user requires prior knowledge of the types of genomic resources available, source of availability, methods to process the raw sequence, computational algorithms and tools to extract and visualize the information, primer designing, and deposition of the data for public access.

### 2.2. Genomic Resources for SSR Data Mining

The data mining approach is a byproduct of several genome sequencing projects. Sequences generated from these projects are made available in databases in several forms, such as whole genome sequences (WGSs), bacterial artificial chromosome (BAC) and P1-derived artificial chromosome (PAC) clones, genome survey sequences (GSSs), pseudomolecules, scaffolds, BAC end sequences, cDNA, expressed sequence tags (ESTs), unigenes, and candidate gene sequences. Depending on the availability and choice, these sequences can be downloaded and used for SSR data mining. DNA from a desired organism is fragmented, integrated into cloning vectors such as BACs, and transformed into bacteria for genome sequencing projects. The entire collection of DNA fragments representing the genome of an organism, integrated into these vectors, constitutes BAC libraries. Microsatellites are more abundant and comprise lengthier repeats in the BAC library. This is significant since the allelic diversity of SSR loci is positively correlated with the repeat number [14]. SSR data mining is also conducted from BAC end sequences, which are terminal DNA sequences of a BAC clone starting from the cloning vector and reading into the insert in both the forward and reverse directions. BAC end sequences provide a glimpse of an un-sequenced genome’s sequence composition and organization [15]. SSRs derived from these BAC end sequences are called BAC end-derived SSRs (BES SSRs). The development of BES SSRs is advantageous since they serve as anchor points between physical and genetic maps. They have potential applications in map-based cloning, genome sequencing projects, and genetic and physical mapping studies. 

Another repository for SSR data mining constitutes expressed sequence tags (ESTs). A vast collection of EST data is available in several species even where genome sequencing projects have not been initiated. These ESTs have been extensively exploited for SSR data mining from the coding regions of the genome [11,16,17,18]. A major problem associated with using ESTs is their redundancy, which leads to the identification of multiple markers at the same locus. To overcome this limitation, overlapping ESTs are clustered to identify a unique singleton known as unigene, and SSRs are mined from these unigenes. In some cases, candidate genes with known functions have also been searched for repeat motifs. This is particularly useful for direct allelic selection in marker-assisted breeding programs. For instance, novel microsatellite markers have been developed from the candidate genes encompassing quantitative trait loci (QTL) associated with zinc and iron content in rice [19]. Similarly, novel salt- and heat-responsive candidate gene-based SSRs have been developed in wheat and rice through in silico mining [20,21,22,23]. Exome capture that targets the whole collection of exons in a genome is an emerging source for identifying genic SSRs. Since these genic microsatellites are from conserved coding regions, they are more preferred for cross-transferability across a group of closely related species. They are a potential tool for comparative mapping and syntenic studies [18,24]. However, for the same reason, genic SSRs also exhibit a low level of polymorphism compared to SSRs obtained from the whole genome.

Genome-wide SSRs can be extracted from scaffolds, pseudomolecules, and genome survey sequences found in numerous sequenced genomes, including those of *Arabidopsis*, humans, rice, and tomato. Scaffolds symbolize an extensive compilation of genome sequences consisting of numerous BAC contigs arranged sequentially in an overlapping tile. In contrast, pseudomolecules represent complete chromosome sequences with gaps to a specific degree. GSSs represent a collection of unannotated, short, single-read nucleotide sequences, including random survey sequences, clone end sequences, and exon-trapped sequences, available in the GenBank division of the National Center for Biotechnology Information (NCBI). These resources are especially helpful when many markers need to be developed from a specific genomic region or chromosome for fine mapping and saturating genetic maps.

**Figure 1:**
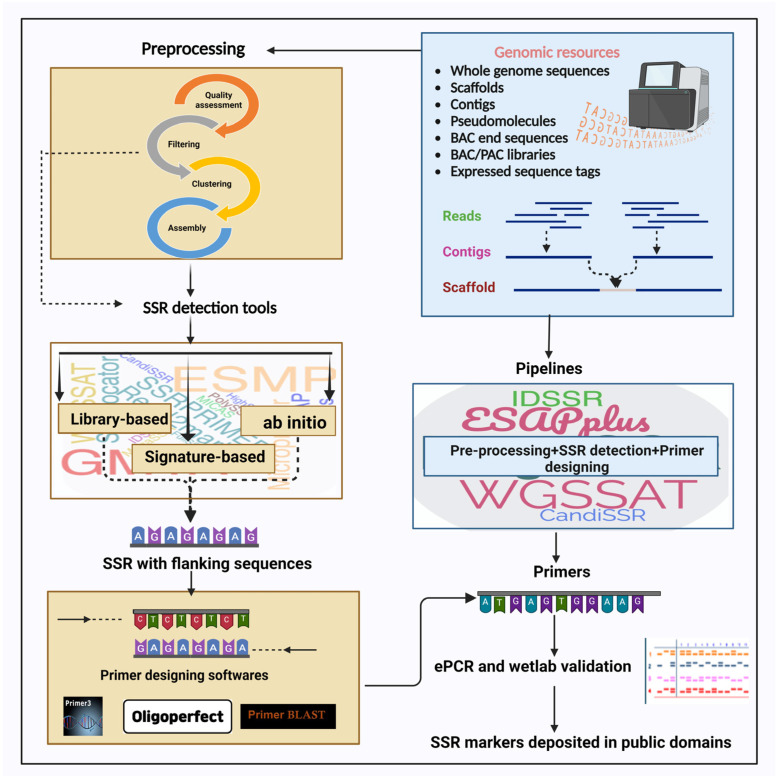
The workflow of SSR data mining and primer designing from genomic resources. Briefly, a wide array of genome sequences made available through library construction and sequencing platforms are subjected to a series of preprocessing steps; clean sequences are searched for SSR motifs using algorithms implemented in SSR detection tools. The repeat motifs with flanking sequences are used as input for primer designing using primer designing software. The primers are then validated and made available as genetic markers. Alternatively, user-friendly pipelines integrating several computational tools that can produce the same output in the form of primers are available for reliable and robust SSR marker development from scratch.

Presently, whole genome sequences derived from high-throughput sequencing platforms are soaring as rapidly expanding genomic resources are utilized for extracting SSRs. Before 2010, the predominant sequencing platforms utilized for short-gun genomic library analysis were 454 GS and Illumina, which effectively facilitated the discovery of SSRs [25]. Illumina sequencing is less expensive, consuming only a fraction of the cost required for the 454 GS platform [26]. The 454 sequencing platform was favored when extended read lengths were necessary. This criterion is crucial in primer design, as it dictates the presence of adequate flanking sequences that contain high-quality PCR priming sites. An additional benefit is that it is possible to precisely determine the number of repeats from 454 reads. However, while using Illumina paired-end (IPE) sequencing, it is impossible to extract the exact number of repeats for many IPE loci [27]. This is because the IPE library insert size is larger than the combination of two paired lengths, and therefore, the SSR loci can extend into the intervening portion that is not covered by both reads [26]. PacBio and nanopore, examples of third-generation sequencing technologies, have enabled the simultaneous perusal of lengthy paragraphs from the genome book. Among these, HiFi sequencing is emerging as the gold standard in the sequencing industry, providing extended reads with high accuracy [28]. The utilization of pangenomics techniques, which are currently prevalent in numerous model organisms, has created opportunities to simultaneously analyze the allelic diversity of SSR motifs and the core genome [29]. Various genomic resources have been used recently for SSR data mining in several plant species (Table 1 and Figure 1).

### 2.3. Databases for Genomic Resources

Public databases provide consolidated access to all available genomic resources. Universal databases contain diverse sequence information from various organisms. GenBank is a universal database that harbors the most extensive compilation of publicly accessible DNA sequences and protein translations. The NCBI maintains this database in partnership with the European Molecular Biology Laboratory (EMBL), the data library of the European Bioinformatics Institute (EBI), and the DNA Data Bank of Japan (DDBJ). The GenBank database has been recently enriched with around 2.9 billion nucleotide sequences representing 504,000 formally described species [60]. GenBank stores its nucleotide data in distinct databases designed explicitly for core nucleotides, ESTs (dbEST), GSSs (dbGSS), and STSs (dbSTS). For ongoing genome sequencing initiatives, species-specific databases provide detailed information exclusively about a single species. Databases with genomic resources specific to plants, insects, microbes, humans, and mice are also available. 

### 2.4. Preprocessing of Raw Sequences

Before beginning the repeat motif search, genomic sequences accessed and downloaded from multiple databases must be assessed for their sequence quality. A clean sequence is necessary for an efficient search. However, some genomic resources represent error-prone and poor-quality sequences and must be preprocessed before data mining. Further, low-complexity portions, vector sequences, and contaminations must be eliminated from the sequences. Preprocessing consists of three phases: cleaning, clustering, and assembly (Figure 1). Previously, tools like PHRED [61] were used for base calling; pairwise alignment tools like cross_match [62], Fasta [63], and BLASTN [64] were used to identify vector sequences and other contaminants against a non-redundant database like Univec; and tools like Seqclean [65], Simple [66], and DUST [67] were used to remove low-complexity regions. Currently, a combination of online tools such as Trimmomatic [68] for adapter trimming and filtering, Cutadapt [69] for adaptor sequence removal, and FastQC [70] for assessing read quality are popularly used resources for cleaning FASTQ sequence files. AfterQC [71] and Fastp [72] were developed to integrate all these tools into a single pipeline. AfterQC, written in Python, is slow while processing huge fastq files. On the other hand, Fastp, designed in C/C++ with multithreading functionality, is an ultra-fast sequence cleaning tool capable of handling single and paired-end reads and lengthy reads generated by the PacBio and Nanopore sequencers. 

Following sequence quality assessment, ESTs must be further processed to avoid redundancy. The overlapping readings from the same gene transcript are combined into a single gene index class by employing stringent or loose clustering techniques [73]. In stringent clustering, the initial fidelity is high, but the data coverage is low and vice versa in the case of loose clustering. EST clustering can be conducted using template sequences or “seeds”, referred to as supervised clustering, or, without any prior knowledge, referred to as unsupervised clustering. The generated gene indices are stored in databases. Three major gene indices include the TIGR (The Institute for Genome Research), STACK (Sequence Tag Alignment and Consensus Knowledge Base), and Unigene. TIGR gene indices use stringent and supervised clustering to obtain short consensus sequences. Here, GenBank CDS, mRNA, and expressed transcript data from the expressed gene anatomy database (EGAD) are used as reference “seed” sequences or templates [74]. STACK leverages loose and unsupervised clustering to obtain a more extended consensus sequence [75]. Unigene blends supervised and unsupervised clustering approaches with varying levels of stringency by using mRNA and coding sequences from GenBank as reference sequences. The TIGR and Unigene indices use pairwise alignment for clustering, while STACK differs from these indices by employing a word-based clustering approach. 

After clustering, sequences within each cluster are aligned to build an assembly, and overlapping segments are merged to potentially reconstruct the transcript’s single, lengthy original sequence. This consensus sequence is referred to as a singleton. Clustering and assembly can be conducted using programs like CRAW [76], CAP3 [77], Phrap [62], TGICL [78], Sequencher, and BAG [79]. The preprocessing step reduces the size of the data set to be analyzed, solves the problem of redundancy, and provides a high-quality sequence for SSR data mining. However, when high-quality, non-redundant genome sequences are available in the databases, they can be used directly for repeat motif searching without being subjected to the preprocessing step.

### 2.5. Computational Tools and Algorithms for SSR Data Mining 

The initial method to analyze a sequence for repeats was by visual inspection. This approach might work for a limited number of sequences. However, automated computational methods have evolved quickly due to the robustness of the data sets to be handled. Any repeat finder program consists of three components: the output compartment, the filter component, and the detecting unit. The search algorithm, which affects the program’s overall time and space efficiency, is housed in the detection unit [80,81]. The algorithm is a set of heuristics and calculations that survey the input data for specific types of repeat patterns. Based on the results of this analysis, optimal parameters are defined and applied across the entire data set to extract the desired kind of repeats. Different tools use either a single algorithm or a combination of algorithms for the repeat finding process. In the filtering step, repeat candidates are subjected to a screening procedure to identify and eliminate redundancies. The output provides a comprehensive report on the type and number of repeats detected and their location. Some tools include graphical representations of the report for easy visualization and understanding.

### 2.6. Algorithmic Approaches

Algorithms developed to date use various ways to find and extract repeats. Repeat detection can be conducted by hunting for novel and unidentified repeat motifs with no prior knowledge or using reference sequences based on previous knowledge of the repeat motifs and their regular expression patterns/signatures. This goal can be addressed using a simple heuristic or extensive combinatorial approach. In the statistical/heuristic approach, short window sizes are specified to identify probable micro-repetitions in a given DNA segment and connect them to longer repeats. In the combinatorial approach, a given sequence is dissected into sub-sequences and compared for detecting repeats [82]. Depending on the type of repetitive architecture to be detected, suffix trees, suffix arrays, the hamming distance model, and the edit distance model have been used to search and store repeat strings. Based on the detection process, repeat finding algorithms could be broadly classified into library-based, signature-based, and ab initio methods (Figure 1). These methods have been reviewed in detail by Bergman and Quesneville [83], Saha et al. [84], and Lerat [85].

### 2.7. Library-Based Methods

In this method, input sequences are searched against a curated library containing reference repeat sequences. Currently, Repbase is the most extensively available curated repeat library with sequence information from several eukaryotic genomes, including human, *Arabidopsis*, rodent, Drosophila, and zebrafish genomes [86]. The tandem repeats database (TRDB) is another repeat library that provides information for about 22 sequenced genomes [87]. However, for organisms that are not included in the existing repeat libraries, new library files can be created from scratch by an ab initio approach using programs like Recon [88], Repeat Scout [89], or RepARK [90]. For example, Stein et al. [91] created a repeat library for *Caenorhabditis elegans* and *C. briggsae* using the Recon program. Repeats are identified based on the degree of homology shared between the library repeat sequence and the query sequence. Similarity scores are generated based on the length and number of gaps in the query and library sequences. When the score exceeds a predefined minimum threshold, the region of the query sequence is considered to harbor the repeat motif. To make this comparison, search engines are required. The most popular search engines employed include cross_match [62], BLAST [92], and Wu Blast (http://blast.wustl.edu; http://genetics.bwh.harvard.edu/msblast/readme.html, accessed on 24 June 2024). 

### 2.8. Signature-Based Methods

This method searches a query sequence for sequence motifs and spatial arrangements characteristic of a particular repeat group. Unlike library-based tools, this warrants prior knowledge of specific repeat types. Signature-based tools employ a heuristics approach.

### 2.9. Ab Initio Approaches

These algorithms detect repeat elements in a query sequence without prior knowledge of repeat motifs or reference to repeat libraries. These methods identify short sequences that occur multiple times in a sequence, using various approaches such as self-comparison approaches, enumeration of k-mers, spaced seed techniques, dot matrix, and periodicity approaches. 

#### 2.9.1. Self-Comparison Approaches

This is a similarity-based searching method, where the uncharacterized DNA sequence is queried against itself using nucleotide–nucleotide blast modules such as BLAST and Wu BLAST to identify clusters of similar sequences.

#### 2.9.2. Enumeration of K-Mers

The k-mer approach is a word counting approach where repeated occurrences of small words known as k-mers are searched. Here, the input DNA sequence comprising A, G, C, and T is considered as a character string of length n. A repeat motif of length k is regarded as a substring of this DNA sequence. Since the DNA sequence comprises A, G, C, and T, there are 4 K possible words of length k. The value of K is determined based on the genome size or the length of the input sequence (n), using the formula K > log4(n). Based on plant genome size estimates, the value of K for indexing assembled plant genomes is estimated to be between 12 and 19 [93]. Within this range, there is a significant increase in the number of unique k-mer sets identified. As the value of K increases beyond this threshold, the sensitivity of the repeat detection and the resolution of the k-mer set decreases [94]. Hence, tools employing the k-mer algorithm should use a compact and efficient representation of substrings for fixing the k-mer size. Once all the repeated exact k-mers exceeding the predefined length threshold have been identified, initial clusters could be built using a suffix tree data structure or fixed length k-mer approach. This primarily helps to reduce time and space complexity issues while handling large data sets [84].

#### 2.9.3. Spaced Seed Approaches

This is an extension of the k-mer approach. While k-mer approaches search for perfectly identical matches, spaced seed algorithms conduct searches for imperfect matches by allowing indels and substitutions to be tolerated in the seed sequence up to a certain threshold level [95,96]. These spaced seed approaches increase the sensitivity and speed of the searches compared to k-mer approaches.

#### 2.9.4. Visualization Approaches

This method is based on direct inspection by human eyes and was one of the earliest and simplest approaches. Later, color-coding algorithms were developed, which assign a color to each of the four bases and display the entire sequence in columns of different widths [97,98]. Although this enables the easy identification of longer and less identical tandem repeats, there is difficulty in viewing large data sets. Another method is the dot plot technique, wherein the sequences are plotted against themselves, and the repeat motifs are visualized as repeat graphs (e.g., Dot plot and Adplot).

#### 2.9.5. Periodicity-Based Approaches

Genes are read as three-letter codons. These exhibit three base periodic signals, which light up as spectral peaks in a DNA power spectrum analysis. Hence, power spectrum techniques, such as the Fourier transform, short-time periodicity transform, periodic subspace decomposition, and correlation functions, have been extensively used to detect this periodicity in genomes and locate the protein-coding regions in the DNA sequence. This concept has further been extended to identify repeat patterns in the genome sequence since tandem repeats also possess the characteristic of a periodic signal [99,100].

Rather than viewing DNA sequences as alphabetic strings, this approach converts sequences into digital signals and is considered a time series. High-intensity peaks in the power spectrum of the sequence represent candidate repetitive elements, but this depends on the type and length of repeats. Many perfect tandem repeats exhibit a strong signal, and the intensity degrades with repeats interrupted by substitutions, insertions, and deletions [100]. The entire sequence is searched using a sliding window for elements similar to the candidate repeats. Pattern structures, word, and distance similarities are used to determine significant periods within a region [101]. Earlier algorithms employing a periodicity approach focused on tandem repeats with short patterns, which were eventually scaled up to detect long patterns. Each of these algorithmic approaches has its advantages and limitations. For instance, on a small scale, all repeat analyses can be conducted quickly using an ad hoc combination of traditional tools. However, combinatorial methods become exhaustive when the data size increases to whole genome analysis. Heuristic search methods are advantageous in reducing the time complexity but are inadequate when the presence or absence of repeat elements needs to be determined with certainty [102]. Hence, the objective of the search is to decide on the algorithm and, in turn, the computational tools for analysis.

The size of genomic resources available for data mining ranges from a few hundred base pairs to a whole genome, constituting several million bases. Several computational tools are available to analyze the exhaustive range of data sets. However, an efficient computational tool used for data mining should satisfy four essential criteria as described by Kurtz et al. [102].

(i) Efficiency: locating repeats in linear time and space. The tool’s memory space and run time to handle large data sets should be linear to sequence length.
(ii) Flexibility and significance: the ability of the tool to identify all possible kinds of repeats, viz., perfect, imperfect, compound, and palindromic repeats.
(iii) Interactive visualization: a user-friendly web interface that could provide an overview of the input sequence and a detailed description of the repeat elements.
(iv) Compositionality: the tool should provide a simple interface to enable composition with advanced analysis tools.

Advances in bioinformatics have contributed to the speedy development of several repeat finding software tools and pipelines since 1994. The available tools satisfy one or more of the criteria mentioned above. The features of some commonly used simple sequence repeat finding tools are discussed below. 

##### Sputnik

This program implemented the first combinatorial approach to identify microsatellites based on repeat size [103]. It accepts input sequence files in fasta/multiple formats. A recursive algorithm searches for 2–5 bp repeat patterns, and a scoring system calls each SSR. The algorithm scans one pattern size at a time detects perfect, imperfect, and compound repeats and returns the output in tabular format. Modified versions of Sputnik, such as Sputnik I and Sputnik II, have also been developed [104].

##### Repeat Masker

A repeat masker is a library-based tool to identify and annotate repetitive elements in DNA sequences and mask them for further analysis [105]. The user must select an existing repeat library, such as Repbase, or generate a new one. Repeat masking can be accessed through a command line or web-based interface. The web repeat masker can analyze sequences shorter than 100 kb. In comparison, the command line version is suitable for sequence sizes exceeding the 100 kb limit and provides more choices for user-defined options. Sequence comparisons are generally performed using the cross_match search engine. However, the time to analyze more extensive sequences, such as the whole chromosomes/genomes of highly evolved species, is significantly longer. In such cases, cross_match can be replaced by another search engine, WuBLAST, for faster processing, where a 30-fold processing time could be reduced [106]. However, using WuBLAST for quick analysis also has limitations, such as lesser sensitivity in masking low-complexity repeats and the accuracy of the results not being assessed [107].

##### TRF

Tandem Repeat Finder (TRF) works on a probabilistic model to detect very large SSRs. An ab initio method called the k-tuple matching algorithm is used to detect perfect, imperfect, and compound SSRs. The analysis component provides an alignment for each candidate and a summary of statistics. The efficiency of this tool was tested on four sequences ranging up to 700 kb size including intron 1 of the human fratoxia gene responsible for triplet repeat disease Friedrichs ataxia, human beta T cell receptor locus, and on yeast chromosomes [108].

##### Reputer

Reputer offers an efficient solution for an exhaustive repeat analysis of genomes. Reputer uses the ab initio approach to identify repeats directly from the nucleotide sequence in a non-heuristic way. It comprises a search engine, REPfind, and a visualization component, REPvis. REPfind employs the k-mer search algorithm. Besides the identification of degenerate perfect repeats, REPfind also detects palindromic repeats. Gusfield’s algorithm is used to compute maximal exact repeats. The maximal mismatch repeat (MMR) algorithm and maximal differences repeat (MDR) algorithm are used to calculate degenerate and compound repeats. The search engine output is displayed as a repeat graph by REPvis that provides a user-friendly interface for examining repeat structures in the genome [102].

##### SSRIT

The SSRIT (Simple Sequence Repeat Identification Tool) is a semi-automated tool that uses regular expressions to identify SSR patterns in fasta-formatted file sequences [31]. It can be used to mine perfect repeats in different types of genomic sequences varying in size from several hundred nucleotides to 1 MB of long contigs assembled from fully sequenced BACs and PACs. Two to ten base repeat motifs are identified, eliminating mononucleotide repeats. This tool was first used for mining perfect repeats in rice genome sequences. A modified version of the SSRIT is available as CUGISSR.

##### TROLL

TROLL stands for Tandem Repeat Occurrence Locator. It is an open-source program that uses a modified Aho–Corasick (AC) algorithm to detect perfect microsatellite motifs up to five base pairs. TROLL comprises a preprocessing (PP) module wherein the user has to provide an input file with a list of motifs/patterns to build the keyword ‘TREE’. This is followed by the AC module, which considers the query DNA sequence a text string. The text string is scanned one by one to match the predefined patterns/motifs listed in the input file of the PP module. The matching technique is similar to a bibliographic search. Whenever a match is found, a buffer control module is executed to keep track of motif occurrences and to avoid missing repeats. The program produces a flexible output format that can be easily integrated with other analysis tools. The limitation of this tool is the unfriendly command line interface [109]. Moreover, it is unsuitable for handling and processing large sequence data sets as the tree consumes larger memory space [110].

##### MISA

MISA, which stands for Microsatellite, is a Perl script-based tool for SSR identification. It mines for both perfect and compound SSRs using regular expression searches. Input files are to be provided as fasta-formatted sequence files. The user has to define the unit size and the minimum number of each repeat. The output file provides the summary statistics of the identified repeats. The utility of this tool was first demonstrated in barley EST sequences [111]. The tool has been recently extended as a web-based application (MISA-Web) with improvements in compound repeat detection and display formats compatible with downstream analysis [112].

##### Poly

This program was developed by Bizzaro and Marx [113]. This Python script-based computer program tracts SSRs in a given sequence using sliding windows of any size. However, rather than finding repeats, the emphasis is more on the quantitative analysis of SSR tracts in the sequence population analyzed.

##### TRA and E-TRA

Tandem Repeat Analyser (TRA) employs two different algorithms independently: exact and inexact modules for repeat detection. Both these modules have their own advantages and limitations. The exact module uses a string-matching algorithm and is fast with several user-defined options but misses inexact repeats. The inexact module captures all exact and inexact repeats but does not permit the user to search for a specified motif length and repeat number. The algorithm employed in STRING [114] combined with a compound repeat finding option is incorporated for the inexact module. Another program, Exact Tandem Repeat Analyser (E-TRA), uses one of the algorithms of TRA [115] for identifying exact tandem repeats and non-simple repeats like compound imperfect and extended compound repeats [116]. Both programs provide knowledge on SSRs mined from sequences derived from different tissues/organs or development stages that would be useful for comparative studies on the expression, regulation, and evolution of repeats. TRA has been demonstrated using the EST sequences from *A. thaliana*, *A. lyrata*, and *A. halleri* sub sp. *halleri* [115], while E-TRA was demonstrated using human EST sequences [116].

##### ATRhunter

ATRhunter employs a string-matching algorithm aimed at identifying more approximate tandem repeats (ATRs) with long motifs. This feature is attributed to its ability to identify the similarity between larger regions, tolerating small mismatch regions within larger fragments. This method employs a two-phase algorithm, viz., a screening phase and a verification phase, to identify windows of high similarity and their adjacent sequences. In the screening phase, an iterative algorithm performs a candidate repeat motif search using a sliding window approach. These candidates are subjected to an alignment scoring test for identifying desired ATRs, and overlapping ATRs of the same motif length are filtered in the verification phase [117].

##### SSR Scanner

SSR scanner is a Perl script-based tool that analyzes large sequences with less running time. It searches repeats of any length using a dictionary approach. This tool mainly aims to scan the genome for repeats and reports the distribution and exact chromosome location of each microsatellite in the genome. The utility of this tool was demonstrated using the *A. thaliana* genome [118].

##### Tandemswan

The main focus of the Tandemswan tool is the identification of fuzzy tandem repeats, which are found in the regulatory regions of eukaryotic genes and interact with transcription factors. Based on the probabilistic model, the periodic signals representing candidate repeats are captured initially using auto-correlation analysis. The identified candidates are then filtered using statistical weights. Mono- and dinucleotide motifs will be missed since this algorithm is designed to detect tandem repeats with a period of three and its multiples. This tool was used to identify short FTRs (3–24 bp) in the genomes of *Drosophila melanogaster* and *D. pseudoobscura* [119].

##### OMWSA

OMWSA stands for Optimized Moving Window Spectral Analysis, a detection and visualization tool for tandem repeats. This periodicity-based approach employs an auto regressive model combined with a moving window spectral analysis. Compared to traditional Fourier transform spectral methods, it produces fewer artifacts and can more reliably identify repeats that undergo excessive mutation [99].

##### SCIROKO

SCIROKO stands for SSR Classification and Investigation by Robert Kofler [120]. This comprises two modules—an SSR search module and an SSR statistics module. The search module is based on a scoring system incorporating the length of microsatellites. Five search modes are available, three for perfect repeats and two for mismatched and compound repeats. In the case of the ideal SSR search modes, repeat detections are based on either a specified number of repeats or the microsatellite length. In the mismatched search mode, the perfect SSRs serve as reference seeds and are iteratively extended in both the 5′ and 3′ directions, tolerating mismatches in indels and substitutions. This algorithm is comparatively faster than imperfect repeat finding programs like Sputnik and TRF. SCIROKO’s SSR statistics module is the first search tool that allows for a systematic survey of associated repeats.

##### JSTRING

JSTRING is a Java version that implements a heuristic algorithm similar to STRING. This tool emphasizes the visualization of the tandem repeats at a glance. It presents a rich interactive user interface with a graphical display of results. Nucleotides are represented as color codes. Using the color-coding approach, the graphical page displays the sequence bands, tandem repeats, and the consensus sequence. It is fast and efficient, wherein the run time for analyzing a sequence length greater than 4 Mbp lasted for five to twenty minutes, depending upon the parameter specified [121].

##### FAIR

FAIR stands for Finding All Internal Repeats. Rather than simple sequence alignments, it utilizes the concept of dynamic programming to identify repeat motifs in nucleotide and protein sequences. The memory required for the program is equal to the memory necessary to store the repeats, and thus, the algorithm runs well for extremely large sequences. The program is implemented as a web-based computing engine, ‘Identseek’. Identseek produces a comprehensive and transparent output by displaying the number of repeats with the start and end positions. The efficiency of the algorithm was tested on the plasmid pYV from *Yersinia pseudotuberculosis* [122,123].

##### CGSSR

The CGSSR (Comparative Genomics for SSR discovery) search tool utilizes a two-phase algorithm, consisting of an auto-correlation phase and an overlapping adjacent phase. In the auto-correlation phase, it is assumed that a repeated substring with a basic pattern exists in the query sequence. Using these as seeds, a frameshifting and matching process is applied to detect the repeat pattern. In this way, all possible mono- to hexanucleotide repeats are identified as candidates. The candidates are then subjected to the overlapping adjacent phase, wherein the overlapping records are verified, and a filtering step removes redundant patterns. Perfect repeats are then used as input sources in the subsequent modules to identify imperfect repeats [124].

##### TReKS

TReKS is an ab initio program that identifies tandem repeats using the K-means clustering algorithm (Treks). This program is implemented in Javascript and has a built-in GUI interface. This tool is linked to the protein repeat database and is mainly tuned for the large-scale identification of repeats in protein sequences. However, the same version can also be applied to nucleotide sequences, which require the optimization of certain parameters [125].

##### MfSAT

MfSAT stands for Multi-Functional SSRs Analytical Tool. This tool specifically identifies SSRs in viral genomes, including DNA/RNA sequences. It uses regular expressions for detection, and the algorithm is the same as IMex. The algorithm uses two independent parameters, the maximum motif and minimum repeat number, to detect mono- to hexanucleotide repeats. Additionally, it can detect codon repeats and report the corresponding amino acid. The output comprises a list of SSRs, repeat number, their abundance, and genomic location [126].

##### PAL Finder

This ab initio tool aims to extract SSRs from sequence reads generated via high-throughput sequencing platforms such as 454 read and Illumina. One of the main reasons for identifying SSRs is to study their allelic length variation. Although several SSRs could be identified, the flanking sequences may be short and not amenable for designing primers due to the secondary structure formation and low-complexity regions. The Illumina and 454 platforms produce sufficiently longer read lengths. This enables the identification of reads containing SSRs in the first step. Subsequently, the flanking sequences of candidate reads are examined for high-quality PCR priming sites to facilitate effective primer design. This results in the identification of a ‘potential amplifiable locus’ (PAL). Stringent filtering criteria produce a set of “Best PALs”. Written in a Perl script version, the tool identifies perfect 2–6 mer repeats, and the efficiency of this tool was demonstrated in Burmese python and two bird (Gunnison Sage-grouse and Clark’s Nutcracker) genomes [26].

##### GMATo

The Genome-wide Microsatellite Analysing Tool was developed for in silico SSR prediction from genomes of any size using a regular expression pattern [127]. Long DNA sequences are chunked into fragments, and repeat motifs from each chunk are identified and combined using the greedy matching algorithm. It uses the Perl language for SSR identification and statistical analysis and Java script for graphic interface. The advantage of this software over the previous tools is that it can be run on multiple platforms, can process multi-fasta files with lesser computing memory, and provides a user-friendly graphical and command interface. The validation of the GMATo was conducted using the published genome sequence of foxtail millet [127]. However, only perfect repeats can be mined in this version, and an additional script is required to identify long compound imperfect repeats in the output.

##### ProGeRF

While most of the SSR data mining tools have been designed to extract the repeat sequences from DNA sequences, the Proteome and Genome Repeat Finder (ProGeRF) is the first tool to detect repeat motifs from both proteome and genome data sets [128]. It utilizes a sliding window method and the concept of SSAHA (Sequence Search and Alignment by Hashing Algorithm) to create a hash table. This hash table consists of several single buckets created through circular doubly linked lists. Each bucket consists of repetitive sequence motifs mapped using a predefined hash function. This strategy enables the ProGeRF to address time complexity and efficiently extract perfect and imperfect repeats faster from multi-fasta DNA or protein sequences files. Since this tool does not permit overlaps, the number of repeats identified is less than that of tools allowing for redundancy such as TRF. The program is written in Perl script, uses the jqscript and Jbrowse plugins for tabular and graphical visualization, and runs on the Linux platform. It can be run both as a stand-alone version and also as a user-friendly web tool.

##### SA-SSR

The identification of suffix trees by Weiner in 1973 [129] paved the way for the development of highly memory-efficient suffix arrays (SAs). The SA-SSR tool uses such a suffix and common longest prefix array [O(n)] algorithm to find tandem repeats of any size, including minisatellites and microsatellites from large data sets. The algorithm was evaluated using the whole genomes of *Escherichia coli*, *Caenorhabditis elegans*, *Drosophila melanogaster*, *Zaire ebolavirus*, and chromosome 4 sequences of *Arabidopsis thaliana*. However, this program is non-interactive and can only be run on the Linux platform [130].

##### Kmer-SSR

Kmer-SSR implemented in a C++ program is the first attempt to use the k-mer decomposition approach for SSR identification in linear time. In addition to the Boolean filter array, it provides a series of filter options such as atomicity, cyclic duplicates, minimum SSR length, enclosed SSRs, and specific SSR period sizes to accurately identify perfect SSRs of any specified length [131]. Validation and testing its performance efficiency against previously reported SSR data mining tools using single chromosomes and genome assemblies from six different species, namely *Anolis carolinensis*, *Chlamydomonas reinhardtii*, *Danio rerio*, *Dictyostelium discoideum*, *Physcomitrella patens*, and *Saccharomyces cerevisiae*, indicated that Kmer-SSR was good at reporting all possible SSRs with great accuracy. Despite its multithreaded and robust nature, its slower computational speed compared to heuristic algorithms, inability to identify fuzzy repeats, dependence on the Linux platform, and lack of web interface are some of the limitations of Kmer-SSR.

##### PERF

The Python-based Exhaustive Repeat Finder (PERF) identifies all SSRs (including pentamers and SSRs that end with partial motifs) from large DNA sequences based on direct string comparison to repeat sets [132]. Using a k-mer decomposition approach, all SSRs in a given DNA string are identified in a single iteration step without redundancy. The output is visualized as a stand-alone interactive HTML report. The human chromosome 1 was used as a test sequence to study the performance efficiency of this tool in comparison with other SSR identification tools such as K-MER, SSRIT, MISA, and MRep. The analysis found that the PERF was 3- to 15-fold faster and uses up to 5-fold less memory than the previously existing algorithms tested, indicating its ultra-speed and exhaustive search capacity.

##### SSRMMD

The Simple Sequence Repeat Molecular Marker Developer (SSRMMD) is a rapid, accurate, and flexible algorithm to mine perfect SSRs and identify candidate polymorphic loci from assembled sequences [133]. The algorithm is written in Perl script and uses an improved regular expression strategy with a greedy matching algorithm, similar to the SSRIT and MISA tools for mining SSRs. In addition, it uses a multithreading technology to improve the computational speed. Assembled sequences such as genomes, transcriptomes, and even a single gene in standard fasta format are taken as input for mining SSRs. Two assembled sequences are taken as input for the identification of polymorphic SSRs, and a high-stringency sequence alignment algorithm is used to identify the unique and conserved SSR flanking sequences. Gou et al. [133] tested the program using six genomes of three crops (two rice genomes, two wheat genomes, and two cotton genomes). This tool identifies more novel and polymorphic SSRs with a relatively higher computational speed than regular expression-based algorithms.

##### EasySSR

This web tool, hosted in a Linux server, uses a command line IMex Version based on a string-matching algorithm for the batch mining of perfect and imperfect repeats from large data sets comprising even complete genomes. The program uses Python and Perl scripts for processing large fasta files, automating file conversions, and executing IMex for data mining. The Imex output is processed and stored in the MariaDB database. The data visualization is based on a user-friendly web interface that does not require additional software installations and enables easy interpretations for beginners in SSR data mining. The tool was validated using 54 genomes of *Corynebacterium pseudotuberculosis* [134].

In addition to the above, an exhaustive list of the available tools, system requirements, programming languages, and algorithms for SSR data mining is provided in Table 2.

## 3. Primer Designing

Once repeats are identified and characterized in a given sequence, these repeats can be amplified through PCR for various molecular studies. For this purpose, the repeats, along with a reasonable length of flanking sequence, are extracted and subjected to primer designing (Figure 1). Since successful PCR amplification depends on the selection of oligonucleotide primers, several factors need to be considered while designing primers. For instance, regions highly rich in AT or GC sequences are not amenable to primer designing. Sequences identified as primers should be short, ranging from 20 to 28 oligonucleotides in length, with a GC content of 50–60 percent. The melting temperature of the forward and reverse primers should be the same, and a range from 50 to 60 °C is preferred for SSR primer pairs. The formation of primer dimers and secondary structures, such as hairpins resulting from the complementarity of the primer sequences, make the primers unavailable for amplification reactions, and such primer sequences should be avoided. The manual selection of optimal PCR primer pairs is tedious. Hence, several types of primer designing software such as Primer3 [152], Oligo [153], BatchPrimer3 [154], Primer_BLASTB [155], FastPCR [156,157] (Supplementary Table S1) are available, which analyze the flanking sequence and return a set of suitable primer pairs that fit the default or user-defined criteria. However, when no primers can be identified based on the specified criteria, the user can try to relax various parameters. Most software use the nearest neighbor thermodynamic properties for calculating the melting temperature.

Once primers are designed, they can be analyzed for amplification properties such as melting temperature (Tm), GC content, secondary structures, and self- and cross-dimers. Based on these properties, a rating is assigned to each primer, and a higher rating indicates stability and higher amplification efficiency. Programs such as Netprimer are exclusively developed for this purpose and require just the primer sequence as input.

## 4. Pipelines

SSR data mining and primer designing involve several sequential steps that are performed using different computational and software tools mentioned above. Each of these resources needs to be accessed and processed from different websites. To simplify the process, pipelines have been developed that combine several tools required for sequential analysis, from preprocessing to primer designing (Figure 1). Considering the dense maze of software, it would be easier for a beginner to start the data mining process with pipelines, a few of which are listed below.

**MICAS**

The Microsatellite Analysis Server (MICAS) pipeline is exclusively dedicated to microsatellite analysis in the sequenced genomes of prokaryote and viral genomes. It integrates the database Micdb for microsatellite information on prokaryotes and viruses, W-SSRF for repeat extraction, and AutoPrimer for primer design. In addition to MICdb sequences, the pipeline allows for the analysis of user-submitted sequences. A systematic output is generated through a dynamic HTML program [158,159].

The pipeline is available at http://www.cdfd.org.in/micas.

**SSRPRIMER**

SSRPRIMER combines Sputnik with Primer3 to detect repeats and design primers [160]. In addition, it has an SSR Taxonomy tree server [161], enabling the web-based searching and browsing of different species and taxa for visualization and downloading SSR primers.

**Read2marker**

Read2marker is a set of scripts programmed to handle large data sets. This pipeline accepts input sequence files as chromatogram or fasta format files. It integrates Phred and PHRAP for base calling and assembling the sequence trace data obtained by sequencing both ends of a clone. A newly built SSR identification tool, srchssr2, is integrated into this pipeline. This simple algorithm is efficient in detecting di- and trinucleotide repeat motifs. However, for screening motifs greater than trinucleotides, srchssr2 must be substituted with other sophisticated repeat finding programs. The algorithm also extracts the flanking sequence, which is screened for redundancy using the BLAST program. Primer3 aids primer designing. This tool also integrates TCOFFEE for multiple alignments of unique clones gathered in a group. The utility of this tool was demonstrated in eggplant and pepper [162].

**SSRlocator**

SSRlocator is a Windows-based pipeline that integrates repeat discovery, followed by primer designing and virtual PCR based on programs written in the Delphi language. The repeat detection algorithm resembles the SSRIT and MISA tools. This tool’s performance was validated by analyzing 28,469 full-length, non-redundant cDNA sequences from *O. sativa* for micro- and minisatellites [148].

**PolySSR**

PolySSR is the first pipeline to detect putatively polymorphic SSRs rather than just SSRs. The only requirement here is the availability of sufficient sequence information from different individuals of a species. This pipeline comprises five modules designed for preprocessing, clustering, polymorphic SSR detection, primer designing, and creating a polymorphic SSR database. It integrates various tools such as cross_match and CAP3 for processing the EST sequence, Sputnik for SSR detection, and Primer3 for primer designing. PolySSR has versatile applications when combined with NGS technologies such as 454 sequencing platforms. Especially for non-model organisms, EST sequences from specific tissues of several genotypes can be obtained at a relatively cheaper cost using these 454 reads and directly processed with PolySSR to identify polymorphic microsatellites rapidly. It is used in tomato, potato, rice, *Arabidopsis*, *Brassica*, and chicken [163].

**WebSat**

This web version, written in PHP and Javascript, uses Ajax techniques for a rich user interface. It integrates the TROLL and Primer 3 programs for repeat finding and primer designing. However, this pipeline is not suitable for processing large data sets due to server restrictions [164].

This pipeline is available at http://purl.oclc.org/NET/websat/.

**ESMP**

The EST SSR Marker Pipeline (ESMP) is a user-friendly, web-based EST assembly and annotation pipeline for data mining SSRs from ESTs [165]. The ESMP web interface has been developed using computer languages such as HTML, CSS, JavaScript, and PHP, and MySQL has been used to store data. The pipeline integrates all sequential steps, such as EST preprocessing, clustering, and assembling EST sequences, followed by mining SSRs from assembled ESTs. Several tools, such as cross_match and TrimEst for preprocessing, CAP3 for clustering, and MISA for SSR identification, have been integrated into this pipeline to carry out these processes. The main feature of this pipeline is that it does not require any database or application installation on the user machine. The user can download and input EST sequences in fasta format with .reads extension. If sequence information is available only as a chromatogram file, then the user needs to convert it into a DNA sequence file using a base-calling program such as Phred. The analyzed data can be retrieved for SSR information in the form of an output file with .rar extension.

**HighSSR**

HighSSR is a microsatellite prediction framework exclusively from the raw data generated via NGS platforms. Using the TRF program, the pipeline initially identifies SSR motifs in the given reads. The identified SSRs are assigned to their consensus canonical motifs. During sequencing, when DNA from several samples is multiplexed, sequences from different samples can be distinguished using multiplex identifiers (MIDs). A hidden Markov model is implemented in the program to recognize these MIDs and to assign the reads to the original sample. Sequences from the same sample with shared canonical consensus and flanking regions are grouped into crude clusters. Using these as seeds, candidate repeat sequences are identified in the subsequent rounds of clustering and added to the crude clusters. The crude clusters are then aligned using the program MUSCLE, which generates a multiple alignment guide tree to identify putative loci and sort paralogs. Based on this, a list of SSR loci in decreasing order of repeat motif size and the length of the flanking regions available for primer designing is reported. When reads from multiple accessions of a taxon are made available, loci showing potential polymorphism across individuals can be prioritized for primer designing using Primer3 implemented in the program. These features of HighSSR permit SSR genotyping directly from sequencing platforms on a large scale with greater resolving power [166].

This pipeline is available at http://code.google.com/p/highssr/.

**QDD**

The QDD pipeline written in perl script was developed to automate all the bioinformatic steps required for SSR identification from NGS datasets, starting from sequence cleaning to primer designing [167]. The original version was initially designed for analysing 454 NGS platform reads. The improvised version QDD version 3.1 [168] can handle Ion Torrent, paired end Ilumina sequences and assembled sequences as input, along with several user-friendly options. 

It is freely available at http://www.imbe.fr/~emeglecz/qdd.

**GMATA**

The Genome-wide Microsatellite Analyzing Tool Package (GMATA), developed by Wang and Wang [169], is a multifunctional, one-step pipeline for mining and mapping SSRs in large genomes with great speed and accuracy. This pipeline integrates six Perl script, R, and Javascript modules. These include DNA preprocessing, SSR data mining, SSR viewing, statistical analysis, developing SSR markers, and e-mapping. From a single input sequence file in fasta format, a long DNA sequence is chunked into fragments of appropriate length with overlapping ends. The SSR motifs are identified within each chunk using a regular expression pattern and greedy matching algorithm in Perl script similar to that of the GMATo. The repeat motifs identified in each chunk are combined to generate the SSR information in the original sequence. The primer design was created using Primer3, and amplification was checked using e-PCR. The statistical analysis of the mined SSRs was conducted using both Perl and R scripts. The user-friendly graphical interface involves Java scripts. An added advantage of this software is that it is independent of the platforms and can be run on Windows/Linux operating systems. This pipeline was validated using seven *Nicotiana* genomes and tested in fifteen Poaceae genomic assemblies.

The GMATA is freely available at http://sourceforge.net/projects/gmata/?source=navbar.

**ESAP Plus**

ESAP Plus is a web-based automated computational pipeline for developing SSR markers exclusively from EST data sets. This pipeline, which runs on the Ubuntu/Linux operating system, integrates several scripts (such as PHP, JAVA, HTML, CSS, and inbuilt shell scripts) and software tools that are required for preprocessing, clustering, assembling, SSR mining, and primer designing from EST data sets [170]. The raw EST sequences in multi-fasta files are preprocessed using the integrated Seqclean software, Univec database, and Repeat masker. The clustering and assembly of the high-quality ESTs obtained are conducted using CD-HIT EST and TGCIL to obtain non-redundant EST candidates. MISA and RepeatMasker are used to identify perfect and compound SSRs from these candidate sequences. The EST-SSR sequences are used for primer designing with the help of Bactchprimer3, which incorporates the SSRIT algorithm for filtering SSRs and selecting good-quality sequences for primer design. The output is stored in the ESAP PLUS MySQL database. This pipeline was validated using sugarcane ESTs generated from 26 cDNA libraries.

This tool is available at http://gbp.kku.ac.th/esap_plus/.

**CandiSSR**

Pipelines like polySSR and SSRpoly, which use a cluster-based strategy, help identify polymorphic SSRs only from EST data sets and are unsuitable for handling large genome sequences generated from NGS platforms. The CandiSSR pipeline overcomes this limitation and identifies candidate polymorphic SSRs from multiple assembled genomes and transcriptome data sets [171]. The SSRs within the assembled sequences are identified, and flanking sequences are retrieved and aligned to the reference sequence using BLAST. Low-quality hits are filtered using the Bioperl package. High-quality polymorphic SSRs are finally identified, and primers are designed from the respective flanking sequences. This pipeline integrates the tools like MISA, BLAST, Primer 3, and Clustal W and is automatically implemented in Perl script and also uses BASH script as an additional component. However, this pipeline can only run on the Linux and Unix operating systems. The genome sequence of six rice species (*Oryza* spp.), reference genome sequence of *A. thaliana*, and transcriptome data of four tea species (*Camelia* spp.) were used to validate CandiSSR. The run time varied with the number of assembled sequences and the genome size [171].

This pipeline is publicly available athttps://github.com/xiaenhua/CandiSSR.

**FullSSR**

FullSSR aims to simplify the SSR identification and primer designing process on extensive genomic data generated from NGS platforms. It uses a combinatorial approach involving string searches, library-based detection, and an optimization algorithm to detect perfect and imperfect tandem repeats. However, FullSSR discards imperfect repeats. While it shares functions similar to PAL FINDER, the primer designing uses an integrated software (Bio::Tools::Run::Primer3), which is a modification of the Bioperl package to create an interface with Primer3. The program written in Perl script and implemented using the Unix command line interface can be run either as a genomic analysis pipeline or as a stand-alone program. This pipeline was tested using 2000 rice genome sequences from the *O. sativa* shotgun sequencing project [172].

This tool is available at https://sourceforge.net/projects/fullssr/.

**WGSSAT**

The Whole Genome Sequencing–SSR Annotation Tool (WGSSAT) is an automated annotation pipeline that works on whole genome sequences [173]. Unlike other SSR data mining tools dedicated to repeat motif identification, this versatile graphical interface pipeline integrates tools for predicting genes and noncoding RNA, along with SSR identification and mapping, primer designing, and cross-species amplification. These integrated tools include Augustus for gene prediction; BLAST, RFAM, MIRBASE, tRNASCAN, and Infernal for RNA prediction; Repeat Masker, RMBLAST, and MISA for SSR mining; Primer3 for primer designing; and the Bowtie tool for mapping predicted SSRs to other genomes. The visualization is aided by the Jvenn and JBrowse plugins. It uses Perl script and JavaNet Beans and supports fasta and gff files. This tool was tested using the fugu (*Takifugu rubripes*) genome on the Ubuntu-Linux platform. However, the run time varied depending on the software integrated, the parameters defined, and the size of the genome.

It is freely available at https://sourceforge.net/projects/wgssat-nbfgr.

**IDSSR**

Insertion/deletion (INDEL) simple sequence repeats (IDSSR) is a freely available pipeline for mining polymorphic SSRs in plant and animal genomes and reduces the need for costly and time-consuming marker validation experiments [174]. It is a user-friendly pipeline implemented using Perl and Bash scripts. It is also the first tool to exploit SSR and INDEL markers to identify potential polymorphic SSRs from a single genome sequence. This pipeline integrates BLAST and SSRIT tools to identify SSRs from the reference genome sequence, SOAP indel to identify INDELs from paired-end reads, and Primer3 to design primers from flanking sequences. After several filtering steps, only SSRs containing INDELs are selected as candidate polymorphic SSRs. This pipeline was validated using the Giant panda genome.

IDSSR is freely available at https://github.com/Allsummerking/IDSSR.

**MicroPrimers**

MicroPrimers is a python-based pipeline that integrates trimmomatic, cutadapt, MISA, and Primer3 to process, identify, and characterize SSRs from NGS data, followed by primer designing from a multi-individual microsatellite library. This pipeline also identifies the possible number of alleles and potential polymorphic SSR loci in a population subset [175].

This pipeline is available at https://github.com/FilAlves/micro-primers.

**MegaSSR**

MegaSSR is a web server and stand-alone application that allows for large-scale SSR mining and primer designing at the whole genome and transcriptome levels. It integrates the MISA tool for SSR identification, custom scripts for SSR-based gene annotations, Primer3 and Primersearch for primer designing, and the in silico validation of the designed primers. This pipeline was validated using 35 genomic sequences from model and non-model organisms, as well as 113 plant transcriptome sequences [176].

This tool is available at https://bioinformatics.um6p.ma/MegaSSR.

## 5. Efficiency of SSR Data Mining Computational Tools

SSR data mining has been simplified with an ever-increasing list of computational tools. Although the features of various tools have been enumerated above, for a new user, it is like a black box, and finding the right tool is challenging. No single tool could be an altogether perfect software for detecting all kinds of repeats [85]. The efficiency of the tool depends on the algorithms and search engines employed, flexibility in the parameter settings, filtering ability to reduce redundancy, time and memory space for analysis, the identification of flanking sequences, and user-friendly interfaces and modules available for analysis [80,81,134]. Performance can be best judged by a cross-comparison of several different programs using a standard data set, although it demands a tremendous amount of work [85]. Even while using a common data set, discrepancies exist in the output result of different tools.

A major cause for discrepancies in detecting tandem repeats among various studies is mainly attributed to the parameter settings employed, algorithms, and search engines used. Critical parameters that can lead to an exponential increase or decrease in the tandem repeats detected include the minimum repeat length, period size constraints, minimum score, and alignment weights [80,81,82]. This was illustrated by performing a meta-analysis in yeast, wherein a three-fold divergence was observed in the frequency of microsatellite motifs detected among seven studies [81]. Leclercq et al. [177] analyzed microsatellite motifs in the human X chromosome using five different repeat finding programs viz., Mreps, Sputnik, STAR, TRF, and Repeat Masker. The results showed that Sputnik, TRF, and Mreps showed an alarming increase in the total number of repeats detected, particularly those of smaller lengths, compared to STAR and Repeat Masker. However, the latter two were more stringent for highly degraded repeats. Discrepancies in the output of tandem repeats due to parameter bias were also observed across several eukaryotic genomes while analyzing them using the same five repeat finding tools. Rather than assessing the tools based on the total number of repeats detected as a whole, examining the distribution of repeats detected by period size showed no significant differences between the repeat finding tools [82].

Parameter settings and algorithmic components go hand in hand in the repeat detection process. Hence, if one needs to assess the inherent capability of the tool in the repeat identification process, the parameter bias should be separated from the algorithmic component for comparison. Lim et al. [82] addressed this issue by searching for perfect repeats under default settings and filtering the outputs across seven repeat finding tools that used combinatorial or heuristic algorithms. The performance of the combinatorial and heuristic algorithms was similar, particularly when the influence of minimum length, mismatches, and indels was removed. However, tools using combinatorial algorithms report a marginal excess of repeats over those using heuristic approaches. A similar comparison for algorithmic performance is difficult when imperfect repeat detection is considered. This is because arriving at optimal parameter settings for comparison is complex, and the canonical consensus cannot be defined due to the variations in the degree of degeneration of biological sequences.

Search engines employed by the algorithm play a vital role in the type of tandem repeats being detected. For instance, Repeat Masker is the tool of choice when longer and more divergent repeats are to be detected. Sputnik, SSRIT, and TROLL identify only perfect repeats, while ATR, TRF, Mreps, and TandemSwan are suited to identify imperfect repeats. However, when the input sequence contains N characters, Mreps cannot process the data; hence, these characters must be removed before analysis.

To validate the accuracy of the detected SSRs, Chen et al. [178] compared several existing repeat finding tools for perfect and imperfect SSRs using three randomly selected DNA fragments of 1,000,000 nucleotides from zebrafish, mice, and humans. The tools included Sputnik, TRF, STRING, Mreps, ATRHunter, Msatfinder, TandemSWAN, SciRoKo, IMEx, and CGSSR. Search criteria were manually set to identify ‘Class I’ SSRs with mono- to hexanucleotide repeat motifs and a 20% tolerance ratio to identify imperfect SSRs. However, some tools could not satisfy these criteria. For example, TandemSwan is not programmed to identify mono- and dinucleotide repeats, while Sputnik is programmed to identify SSRs with basic patterns of 25 nucleotides. A comparison of the above tools for perfect repeats showed that TandemSwan, ATRhunter, and Msatfinder did not detect any ideal repeats. Mreps, SciRoKo, IMEx, and CGSSR were more efficient in identifying perfect repeats, while STRING identified the least number of repeats for identical sequences. All ten tools identified imperfect repeats. However, CGSSR was found to be more efficient than all other tools. Using the whole genome chromosomal sequences of *Aspergillus fumigates*, Mathur et al. [179] made a comparative analysis of the repeat finding ability of five publicly available tools, viz., MISA, Msatfinder, Sciroko, SSRserver, and TRF, among which TRF reported the significantly least number of repeats. Similarly, in another study by Wexler et al. [117], ATRhunter identified 35–70% more repeats than TRF. Artifacts can also contribute to an exponential increase in repeat detection. Artifacts can arise mainly due to overlapping repeats. For instance, a more extended repeat with multiple short periods can be identified as a single repeat or multiple repeats with short periods. Tools that do not have a filter option, like T-Reks and inverters, report excess repeats. Tools that allow for filtering (Mreps, ATRHunter, and IMEx) and those that do not allow for repeat search at the same loci (Sputnik) report less to nil overlapping repeats [82].

The next critical factors that decide the efficiency of the tools are the computational time and memory required for analysis. The larger the sequence size and more complex the search, the greater the time and space requirement [180]. The computational time is the minimum for algorithms that detect short and exact repeats, followed by algorithms that detect approximate repeats under the hamming distance model. In contrast, the maximum computational time is recorded by algorithms that detect approximate repeats using the edit distance model [81]. Heuristic algorithms require less time and space compared to combinatorial algorithms. To increase the processing speed, the concept of dynamic programming is being employed in string-matching algorithms. Further, dependency on additional modules in the case of SSR data mining pipelines reduces computational speed [133].

In an earlier study by Castelo et al. [109], TROLL and Sputnik were compared for their execution time to analyze a single chromosome of *A. thaliana*, which was 33 Mb long. While Sputnik required approximately 47 s to complete the task, TROLL accomplished the analysis in 41 s. Wexler et al. [117] reported that TRF was 25% faster than ATRhunter while analyzing the *E. coli* genome for microsatellite motifs. Kofler et al. [120] reported that Sciroko’s performance was considerably faster than TRF, Sputnik, and its modified versions based on sequence analysis from rye, *Saccharomyces cerevisiae*, *Gibberella zeae*, and *O. sativa*. As the sequence size increased beyond 10 Mb, Sputnik failed to generate results.

The motif threshold is another critical factor in deciding the computational speed. Despite supporting multithreading, Kmer-SSR showed poor performance in computational speed compared to the single-threading SSRMMD, owing to inflexible motif thresholds [133]. Although large data sets can be analyzed, in TRA and eTRA, the running time was dependent on the search parameter options and the operating system used. It takes several hours to search for motifs >200 bp when an inexact module is used to analyze multiple files with multiple sequences and is unsuitable for whole genome search. However, these tools find utility for mining repeats in ESTs and small genomes such as bacteria, chloroplasts, and mitochondria, where the genome size is less than one MB.

Using five model species, viz., yeast, fruit fly, zebrafish, mice, and humans, Chen et al. [178] compared the processing time of eight tools, viz., TRF, STRING, Mreps, Msatfinder, TandemSWAN, SciRoKo, IMEx, and CGSSR. Since the genome size of vertebrate species is larger, a single chromosome from each model species was taken for analysis. The required computational time for each algorithm was performed in batch mode and measured in 1/100 of a second. Irrespective of whether the tools were Windows- or Linux-based, auto-correlation- and window slicing-based algorithms such as CGSSR, Imex, SciRoKo, and TandemSwan were found to be fast and efficient compared to other algorithms. EasySSR, an online application that uses the IMEx 2.1 version, was recently developed to process several genomes in one go but had to compromise on the computational speed compared to the stand-alone command line IMEx version [134].

Analyzing various factors involved in the repeat detection process, the choice of tool remains with the user depending on the purpose, input sequence size, the nature of repeats to be detected, and the system configurations available. Table 2 and Table 3 could be used as a reference guide to facilitate the decision-making process in selecting suitable SSR data mining computational tools.

On the one hand, while extensive comparative analysis is being conducted on the performance of a wide array of web-based tools that have been developed, published, and released continuously, on the other hand, links to many of these computational tools cited in articles are no longer functional or available to end users (Table 2 and TE Hub [181]; http://tehub.org accessed on 2 August 2024) raising a growing concern about the time and resources spent on developing these tools, just to become obsolete. Nevertheless, microsatellites mined using these tools are still publicly available in several microsatellite-specific databases for access by the research community. These include general databases for prokaryotes and eukaryotes [87,158,159,182,183,184,185,186,187,188] and organism-specific databases such as humans [189,190,191,192], mouse [193], insects [194,195], fungi [196], viruses [197] and plants [198,199,200,201,202,203,204,205,206,207,208,209,210,211]. Recently, a pan-species microsatellite database (PSMD) has been developed that contains repeats mined from 18408 organisms [212] (Supplementary Table S2).

## 6. Conclusions

Simple sequence repeats are common and widely dispersed across the genome. They have emerged as practical genetic markers due to their molecular characteristics. SSRs are a valuable tool for crop genomics, allowing researchers to solve genetic mysteries, improve breeding programs, and better understand crop evolution. Their adaptability and dependability have made them vital in the agricultural industry. Microsatellites in any species require preceding sequencing information. Library creation was the only option to recover repetitive sequences for a novel species that lacked sequence information. This is tiresome and time-consuming. Alternatively, in silico data mining algorithms provide a cost-effective and rapid method for developing SSR markers. A large volume of sequence data from several sequencing initiatives is a significant resource for SSR data mining. These data must be processed and computationally evaluated to discover and characterize repetitions and design primers. However, the output from many techniques differs, and no single tool is ideal for finding and describing all types of repeats. As a result, the choice of computational tools is determined by the user’s requirements, the algorithms used, and the modules’ speed and flexibility. Managing the appropriate parameter settings presents a significant challenge and necessitates careful attention in utilizing genomic information. The accessibility of command line applications may be limited for biologists who do not possess expertise in bioinformatics. For a new user, “one-stop shop”, user-friendly pipelines that integrate several software tools for preprocessing, repeat identification, primer creation with default settings, and data visualization would be an easy way to obtain insights into data mining methodologies.

## Figures and Tables

**Table 1:** Some recent examples of genomic resources used for developing SSR markers through data mining approaches in plants.

Sequences for SSR Data Mining	Organism	References
PAC	Rice (*Oryza sativa*)	[30,31]
BAC	Tomato (*Solanum lycopersicum*)	[32,33,34]
BAC end	Spinach (*Spinacia oleracea*)	[35]
	Sweet potato (*Ipomoea batatas*)	[36]
EST	Sugarcane (*Saccharum officinarum*)	[37]
	Safflower (*Carthamus tinctorius*)	[17]
	Lemon (*Citrus limon*)	[15]
	Tea (*Camellia sinensis*)	[38]
	Glory lily (*Gloriosa superba*)	[39]
	Siam tulip (*Curcuma alismatifolia*)	[40]
Unigenes	Indian chrysanthemum (*Chrysanthemum indicum*)	[41]
	Tick trefoil (*Uraria lagopodioides*)	[42]
Candidate genes	Wheat (*Triticum aestivum*)	[20,22,23]
	Rice (*O. sativa*)	[19,21]
	Sugarcane (*S. officinarum*)	[43]
	Potato (*Solanum tuberosum*)	[44]
Genomic survey sequences	Peanut (*Arachis hypogaea*)	[45]
	Rye (*Secale cereale*)	[46]
Pseudomolecules	Eucalyptus (*Eucalyptus* spp.)	[47]
	Pineapple (*Ananas comosus*)	[48]
	Eggplant (*Solanum melongena*)	[49]
	Pigeon pea (*Cajanus cajan*)	[50]
Scaffolds	Mandarin orange *(Citrus reticulata*)	[51]
	Carrot (*Daucus carota*)	[52]
WGSs	Rice (*O. sativa*) (PacBio)	[53]
	Maize (*Zea mays*) (PacBio)	[54]
	Cardamon (*Elettaria cardamomum*) (Nanopore and Illumina)	[55]
	Strand sedge *(Carex pumila*) (Illumina and PacBio)	[56]
	Coconut (*Cocos nucifera*)	[57]
	Spinach (*S. oleracea*)	[58]
	Chickpea (*Cicer arietinum*)	[59]

**Table 2:** Features of computational tools developed for identification of simple sequence repeats from genomic resources.

Tool	Algorithm/Detection Method	Script	Platform	URL (Accessed on 24 June 2024)	Type of Tandem Repeats Detected	Reference
Sputnik **	Recursive	C	Windows	http://espressosoftware.com/sputnik/ Updated: https://web.archive.org/web/20060710223631/http://cbi.labri.u-bordeaux.fr/outils/Pise/sputnik.html	Perfect and approximate repeats	[103]
Repeat masker	String matching	Perl	Unix/Linux	www.repeatmasker.org	Perfect, imperfect, and compound repeats	[105]
Tandem Repeat finder (TRF) **	Heuristic: based on K-tuple match and alignments	NA	System independent	c3.biomath.mssm.edu/trf.html Updated: https://tandem.bu.edu/trf/trf.html	Perfect, imperfect, and compound repeats	[108]
Reputer	K-mer approach and suffix trees, Hamming edit distance model	NA	Unix	http://bibiserv.techfak.uni-bielefeld.de/reputer/	Perfect, imperfect, and compound repeats	[102]
Repeat finder	K-mer approach and clustering	NA	Unix/Linux	http://www.genet.sickkids.on.ca/~ali/repeatfinder.html	Perfect repeats	[135]
Simple sequence repeat identification tool (SSRIT) **	Regular expressions and similarity searches	Perl script	System independent	http://brie2.cshl.org:8082/gramene/searches/ssrtool Updated: https://archive.gramene.org/db/markers/ssrtool	Perfect repeats	[31]
ComplexTR *	Seed extension technique and K-length substrings	C++, Perl	NA	http://www.cs.wisc.edu/areas/theory	Variable-length and multiple-period tandem repeats	[101]
POLY	Sliding window approach	Python	Not known	http://bioinformatics.org/poly/	Perfect repeats	[113]
Tandem repeats Occurrence locator (TROLL)	Dictionary approach Aho–Corasick algorithm	C++ (Tcp/Tk script)	Linux	http://finder.sourceforge.net/	Perfect repeats	[109]
Search for tandem repeats in Genomes (STRING) **	Heuristic and auto-alignment search using dynamic programming	C	Unix	http://www.caspur.it/~castri/STRING/ Updated: https://www.parsival.it/STRING/	Perfect and imperfect repeats	[114]
Microsatellite search (MISA)	Regular expression	Perl	System independent	http://pgrc.ipk-gatersleben.de/misa/	Perfect and compound repeats	[111]
Mreps	Mixed combinatorial/heuristic	ANSI C	Linux, SunOS, Digital Unix, Windows	http://www.loria.fr/mreps/	Fuzzy tandem repeats	[136]
Inverted Repeat Finder (IRF)	K-tuple match and alignment score	NA	Windows, Linux, Mac OS	http://tandem-test.bu.edu/cgi-bin/irdb/irdb.exe	Approximate inverted repeats	[137]
Spectral repeat finder (SRF)	Periodicity approach, Fourier transform	Perl	System independent	http://www.imtech.res.in/raghava/srf	Perfect and imperfect repeats	[100]
Search for tandem approximate Repeats (STAR)	Minimum distance length criterion, data compression, and optimization algorithm	NA	Linux, SunOS, Mac OSX, and Windows	http://atgc.lirmm.fr/star	Approximate tandem repeats	[138]
Exhaustive whole genome Tandem Repeat Search (ExTRS)	K-mer and Hamming distance	NA	-	On request from the authors	Variable-length tandem repeats	[139]
Tandem Repeats Analyser (TRA) *	String matching and algorithm similar to STRING	C++	Windows	ftp.akdeniz.edu.tr/Araclar/TRA	Perfect and imperfect repeats	[115]
ATRHunter *	Iterative string matching and dynamic programming	NA	Windows, Unix, Linux	www.bioinfo.cs.technion.ac.il/ATRHunter	Approximate tandem repeats	[117]
Exact tandem repeats Analyser (E-TRA) *	One of the TRA algorithms	C++	Windows	ftp.akdeniz.edu.tr/Araclar/e-TRA	Perfect, imperfect, and compound repeats	[116]
Repeat fetcher *	Pattern recognition, regular expression	Perl	Unix	phoenix.cs.iupui.edu	Perfect repeats	[140]
MsatFinder **	Regular expressions	Perl	Linux	http://www.bioinf.ceh.ac.uk/msatfinder/ Updated: https://web.archive.org/web/20071026090642/http://www.genomics.ceh.ac.uk/msatfinder/	Perfect repeats	[141]
FireµSat/ FireµSat_2_ *	Regular expressions, FA, and Moore machine technology	C++	Windows, Linux	http://www.dna-algo.co.za/downloads.htm	Perfect repeats	[142,143]
Phobos	Exact search	NA	Mac, Linux, Windows	http://www.ruhr-uni-bochum.de/ecoevo/cm/cm_phobos.htm	Perfect and imperfect tandem repeats	[144]
SSRscanner	Dictionary approach based on preselected motifs	Perl	System independent	Available on request from authors	Perfect repeats	[118]
TandemSWAN *	Auto-correlation analysis and statistical weights	C++	System independent	http://bioinform.genetika.ru/	Fuzzy tandem repeats	[119]
OMWSA	Periodicity approach using moving window spectral analysis	NA	NA	http://www.hy8.com/~tec/sw01/omwsa01.zip	Perfect, imperfect, and compound repeats	[99]
Imperfect Microsatellite Extraction (IMEx) **	String matching algorithm and sliding window approach	C	System independent	http://203.197.254.154/IMEX/ or http://www.cdfd.org.in/imex http://www.mcr.org.in/imex/index.html	Imperfect repeats	[145]
SciRoko **	SSR seed extension	C	windows	www.kofler.or.at/Bioinformatics Updated: https://kofler.or.at/bioinformatics/SciRoKo/Download.html	Perfect and imperfect repeats	[120]
JSTRING **	Similar to STRING	Java	System independent	http://bioinf.dms.med.uniroma1.it/JSTRING/ Updated: https://www.parsival.it/JSTRING/	Perfect and imperfect tandem repeats	[121]
msatcommander	Regular expressions	Python	MacOS X, Windows, Unix	http://code.google.com/p/msatcommander/	Perfect repeats	[146]
ReRep (read Repeat) Finder *	Similarity searches	Perl	Linux	http://bioinfo.pdtis.fiocruz.br/ReRep/	Denovo repeat identification in GSS	[147]
SSRlocator *	Similar to MISA and SSRIT	Perl	Windows	http://www.ufpel.edu.br/~lmaia.faem	Perfect and imperfect repeats	[148]
SWELFE	Alignment based on dynamic programming	C	Linux and Mac OS X	http://bioserv.rpbs.jussieu.fr/swelfe	Internal repeats	[149]
TREKS	K-means clustering algorithm	Java	windows	http://bioinfo.montp.cnrs.fr/?r=t-reks	Perfect and imperfect repeats	[125]
FAIR **	Dynamic programming	C++	Web-based	http://bioserver1.physics.iisc.ernet.in/fair/ Updated: http://bioserver1.physics.iisc.ac.in/cgi-bin/fair4/fair/indx.pl	Internal repeats	[123]
TRStalker	Heuristic Edit distance	NA	Unknown	bioalgo.iit.cnr.it	Fuzzy tandem repeats	[150]
Mfsat *	Regular expressions	NA	windows	http://hudacm11.mysinamail.com/hunan.html	Perfect repeats	[126]
PALFINDER	Text search	Perl	System independent	http://sourceforge.Net/projects/palfinder/	Perfect repeats	[26]
GMATo	Regular expression with a greedy matching algorithm	Perl	System independent	http://sourceforge.net/projects/gmato/files/?source=navbar	Perfect repeats	[127]
ProGeRF *	Sequence search and alignment by hashing algorithm	Perl and C	Linux	http://64.79.105.19/ligp/	Perfect and imperfect repeats	[128]
Repeat Analyzer	Knuth–Morris–Pratt (KMP) string searching algorithm	Python	Windows, Linux, Mac OS X	https://bitbucket.org/repeatgroup/repeatanalyzer	Genic SSRs	[151]
SA-SSR	Suffix and prefix array		Linux	https://github.com/ridgelab/SA-SSR	Micro- and minisatellites	[130]
Kmer-SSR	K-mer approach	C++	Linux	https://github.com/ridgelab/Kmer-SSR	Perfect repeats	[131]
PERF	K-mer approach	Python	System independent	https://github.com/rkmlab/perf	Perfect and imperfect repeats	[132]
SSRMMD	Regular expression with a greedy matching algorithm	Perl	System independent	https://github.com/GouXiangJian/SSRMMD	Perfect repeats and polymorphic SSRs	[133]
EasySSR	String matching implemented in IMex	Python and Perl	Linux	https://github.com/engbiopct/EasySSR.	Perfect and imperfect repeats	[134]

* The links to the software in the cited publications are currently not accessible. ** The links to the software in the cited publication have been moved to new URLs.

**Table 3:** A quick reference for suitable computational tools associated with SSR data mining.

Objective	Suitable Computational Tools
Whole genome search for SSRs at a faster pace	Sciroko
Mining for repeats within GSSs	ReREP
Mining for microsatellites in viral genomes	Mfsat
Mining for internal repeats	Swelfe, IRF, FAIR
Mining for perfect repeats only	SSRIT, CUGISSR, TROLL, Sputnik
Mining for perfect, imperfect, and compound repeats	MISA, IMEx, Msatfinder, TRF
Mining for repeats within reads obtained from sequencing platforms	Palfinder
Mining for polymorphic SSR	Palfinder, PolySSR
Mining for fuzzy tandem repeats/VNTR	Tandem swan, ATR hunter, TRF, Mreps, STRING, STAR,
Identification and masking repeats	Repeatmasker, SIMPLE, DUST
Mining for long and divergent repeats	Repeat masker
Mining for short repeats	IMEx, Sputnik
Mining for repeats in both nucleic acid and protein sequences	FAIR, TreKs
Mining for palindromic repeats	Adplot, Reputer, CRISPRFinder
Pipelines	Read2marker, QDD, ESMP, POLYSSR, HighSSR, FullSSR, WGSSAT, IDSSR

## Supplementary Materials

The following supporting information can be downloaded at https://www.mdpi.com/article/10.3390/plants13182619/s1, Table S1: Features of primer designing software; Table S2: Features of web-based resources specifically created for accessing information on microsatellites in various organisms.

## References

[1] The ENCODE Project Consortium (2012). An integrated encyclopedia of DNA elements in the human genome. Nature.

[2] de Koning A.J., Gu W., Castoe T.A., Batzer M.A., Pollock D.D. (2011). Repetitive elements may comprise over two-thirds of the human genome. PLoS Genet..

[3] Liehr T. (2021). Repetitive elements in humans. Int. J. Mol. Sci..

[4] Thakur J., Packiaraj J., Henikoff S. (2021). Sequence, chromatin and evolution of satellite DNA. Int. J. Mol. Sci..

[5] Balzano E., Pelliccia F., Giunta S. (2021). Genome (in)stability at tandem repeats. Semin. Cell Dev. Biol..

[6] Bhargava A., Fuentes F. (2010). Mutational dynamics of microsatellites. Mol. Biotechnol..

[7] Biscotti M.A., Olmo E., Heslop-Harrison J. (2015). Repetitive DNA in eukaryotic genomes. Chromosome Res..

[8] Gemayel R., Vinces M.D., Legendre M., Verstrepen K.J. (2010). Variable tandem repeats accelerate evolution of coding and regulatory sequences. Annu. Rev. Genet..

[9] Lower S.S., McGurk M.P., Clark A.G., Barbash D.A. (2018). Satellite DNA evolution: Old ideas, new approaches. Curr. Opin. Genet. Dev..

[10] Pereira G., Nunes E., Laperuta L., Braga M., Penha H., Diniz A., Munhoz C., Gazaffi R., Garcia A.A.F., Vieira M.L.C. (2013). Molecular polymorphism and linkage analysis in sweet passion fruit, an outcrossing species. Ann. Appl. Biol..

[11] Varshney R.K., Graner A., Sorrells M.E. (2005). Genic microsatellite markers in plants: Features and applications. Trends Biotechnol..

[12] Zane L., Bargelloni L., Patarnello T. (2002). Strategies for microsatellite isolation: A review. Mol. Ecol..

[13] Techen N., Arias R.S., Glynn N.C., Pan Z., Khan I.A., Scheffler B.E. (2010). Optimized construction of microsatellite-enriched libraries. Mol. Ecol. Resour..

[14] Ellison C.K., Shaw K.L. (2010). Mining non-model genomic libraries for microsatellites: BAC versus EST libraries and the generation of allelic richness. BMC Genom..

[15] Hong C., Lee S., Park J., Plaha P., Park Y., Lee Y., Choi J., Kim K., Lee J., Lee J. (2004). Construction of a BAC library of Korean ginseng and initial analysis of BAC-end sequences. Mol. Genet. Genom..

[16] Kalita B., Roy A., Lakshmi P. (2022). In-silico mining and characterization of EST-SSRs for the genetic diversity analysis of lemon. Nelumbo.

[17] Poornima K.N., Shankar R., Ramesh S., Ravishankar K.V. (2023). De-novo development and validation of EST-SSRs in *Moringa oliefera*. J. Plant Biochem. Biotechnol..

[18] Singh K.N., Parveen S., Kaushik P., Goel S., Jagannath A., Kumar K., Agarwal M. (2022). Identification and validation of in silico mined polymorphic EST-SSR for genetic diversity and cross-species transferability studies in safflower. J. Plant Biochem. Biotechnol..

[19] Chandel G., Samuel P., Dubey M., Meena R. (2011). In silico expression analysis of QTL specific candidate genes for grain micronutrient (Fe/Zn) content using ESTs and MPSS signature analysis in rice (*Oryza sativa* L.). J. Plant Genet. Transgenics.

[20] Mehta G., Muthusamy S.K., Singh G., Sharma P. (2021). Identification and development of novel salt-responsive candidate gene based SSRs (cg-SSRs) and MIR gene based SSRs (mir-SSRs) in bread wheat (*Triticum aestivum*). Sci. Rep..

[21] Molla K.A., Azharudheen T.M., Ray S., Sarkar S., Swain A., Chakraborti M., Vijayan J., Singh O.N., Baig M.J., Mukherjee A.K. (2019). Novel biotic stress responsive candidate gene based SSR (cgSSR) markers from rice. Euphytica.

[22] Sharma P., Mehta G., Muthusamy S.K., Singh S.K., Singh G.P. (2021). Development and validation of heat-responsive candidate gene and miRNA gene based SSR markers to analysis genetic diversity in wheat for heat tolerance breeding. Mol. Biol. Rep..

[23] Singh A.K., Chaurasia S., Kumar S., Singh R., Kumari J., Yadav M.C., Singh N., Gaba S., Jacob S.R. (2018). Identification, analysis and development of salt responsive candidate gene based SSR markers in wheat. BMC Plant Biol..

[24] Varshney R.K., Mahendar T., Aggarwal R.K., Börner A. (2007). Genic molecular markers in plants: Development and applications. Genomics-Assisted Crop Improvement.

[25] Zalapa J.E., Cuevas H., Zhu H., Steffan S., Senalik D., Zeldin E., McCown B., Harbut R., Simon P. (2012). Using next-generation sequencing approaches to isolate simple sequence repeat (SSR) loci in the plant sciences. Am. J. Bot..

[26] Castoe T.A., Poole A.W., De Koning A.J., Jones K.L., Tomback D.F., Oyler-McCance S.J., Fike J.A., Lance S.L., Streicher J.W., Smith E.N. (2012). Rapid microsatellite identification from Illumina paired-end genomic sequencing in two birds and a snake. PLoS ONE.

[27] Jennings T., Knaus B., Mullins T., Haig S., Cronn R. (2011). Multiplexed microsatellite recovery using massively parallel sequencing. Mol. Ecol. Resour..

[28] Hon T., Mars K., Young G., Tsai Y.-C., Karalius J.W., Landolin J.M., Maurer N., Kudrna D., Hardigan M.A., Steiner C.C. (2020). Highly accurate long-read HiFi sequencing data for five complex genomes. Sci. Data.

[29] Lu T.-Y., Chaisson M.J., The Human Genome Structural Variation Consortium (2021). Profiling variable-number tandem repeat variation across populations using repeat-pangenome graphs. Nat. Commun..

[30] McCouch S.R., Teytelman L., Xu Y., Lobos K.B., Clare K., Walton M., Fu B., Maghirang R., Li Z., Xing Y. (2002). Development and mapping of 2240 new SSR markers for rice (*Oryza sativa* L.). DNA Res..

[31] Temnykh S., DeClerck G., Lukashova A., Lipovich L., Cartinhour S., McCouch S. (2001). Computational and experimental analysis of microsatellites in rice (*Oryza sativa* L.): Frequency, length variation, transposon associations, and genetic marker potential. Genome Res..

[32] Brake M., Al-Qadumii L., Hamasha H., Migdadi H., Awad A., Haddad N., Sadder M.T. (2022). Development of SSR markers linked to stress responsive genes along tomato chromosome 3 (*Solanum lycopersicum* L.). BioTech.

[33] Geethanjali S., Chen K.-Y., Pastrana D.V., Wang J.-F. (2010). Development and characterization of tomato SSR markers from genomic sequences of anchored BAC clones on chromosome 6. Euphytica.

[34] Geethanjali S., Kadirvel P., de la Peña R., Rao E.S., Wang J.-F. (2011). Development of tomato SSR markers from anchored BAC clones of chromosome 12 and their application for genetic diversity analysis and linkage mapping. Euphytica.

[35] Feng C., Bluhm B.H., Correll J.C. (2015). Construction of a spinach bacterial artificial chromosome (BAC) library as a resource for gene identification and marker development. Plant Mol. Biol. Report..

[36] Meng Y., Zheng C., Li H., Li A., Zhai H., Wang Q., He S., Zhao N., Zhang H., Gao S. (2021). Development of a high-density SSR genetic linkage map in sweet potato. Crop J..

[37] Jiang H., Waseem M., Liu P. (2023). Development of simple sequence repeat markers for sugarcane from data mining of expressed sequence tags. Front. Plant Sci..

[38] Muoki R., Maangi J., Korir R., Bargul J., Kamunya S. (2020). Mining and validation of polymorphic EST-SSR markers for analysing genetic diversity among interspecific hybrids of tea. Int. J. Tea Sci..

[39] Das M., Sahu S.P., Tiwari A. (2020). De novo transcriptome assembly and mining of EST-SSR markers in *Gloriosa superba*. J. Genet..

[40] Taheri S., Abdullah T.L., Rafii M., Harikrishna J.A., Werbrouck S.P., Teo C.H., Sahebi M., Azizi P. (2019). De novo assembly of transcriptomes, mining, and development of novel EST-SSR markers in *Curcuma alismatifolia* (Zingiberaceae family) through Illumina sequencing. Sci. Rep..

[41] Han Z., Ma X., Wei M., Zhao T., Zhan R., Chen W. (2018). SSR marker development and intraspecific genetic divergence exploration of *Chrysanthemum indicum* based on transcriptome analysis. BMC Genom..

[42] Liu C., Zhang M., Zhao X. (2023). Development of unigene-derived SSR markers from RNA-seq data of *Uraria lagopodioides* (Fabaceae) and their application in the genus Uraria Desv. (Fabaceae). BMC Plant Biol..

[43] Divakar S., Jha R.K., Singh A. (2023). Validation of candidate gene-based EST-SSR markers for sugar yield in sugarcane. Front. Plant Sci..

[44] Schumacher C., Krannich C.T., Maletzki L., Köhl K., Kopka J., Sprenger H., Hincha D.K., Seddig S., Peters R., Hamera S. (2021). Unravelling differences in candidate genes for drought tolerance in potato (*Solanum tuberosum* L.) by use of new functional microsatellite markers. Genes.

[45] Zhou X., Dong Y., Zhao J., Huang L., Ren X., Chen Y., Huang S., Liao B., Lei Y., Yan L. (2016). Genomic survey sequencing for development and validation of single-locus SSR markers in peanut (*Arachis hypogaea* L.). BMC Genom..

[46] Li J., Zhou R., Endo T.R., Stein N. (2018). High-throughput development of SSR marker candidates and their chromosomal assignment in rye (*Secale cereale* L.). Plant Breed..

[47] Patturaj M., Munusamy A., Kannan N., Kandasamy U., Ramasamy Y. (2021). Chromosome-specific polymorphic SSR markers in tropical eucalypt species using low coverage whole genome sequences: Systematic characterization and validation. Genom. Inform..

[48] Nashima K., Hosaka F., Terakami S., Kunihisa M., Nishitani C., Moromizato C., Takeuchi M., Shoda M., Tarora K., Urasaki N. (2020). SSR markers developed using next-generation sequencing technology in pineapple, *Ananas comosus* (L.) Merr. Breed. Sci..

[49] Portis E., Lanteri S., Barchi L., Portis F., Valente L., Toppino L., Rotino G.L., Acquadro A. (2018). Comprehensive characterization of simple sequence repeats in eggplant (*Solanum melongena* L.) genome and construction of a web resource. Front. Plant Sci..

[50] Varshney R.K., Chen W., Li Y., Bharti A.K., Saxena R.K., Schlueter J.A., Donoghue M.T., Azam S., Fan G., Whaley A.M. (2012). Draft genome sequence of pigeonpea (*Cajanus cajan*), an orphan legume crop of resource-poor farmers. Nat. Biotechnol..

[51] Jabeen S., Saif R., Haq R., Hayat A., Naz S. (2023). Whole-genome sequencing and variant discovery of *Citrus reticulata* “Kinnow” from Pakistan. Funct. Integr. Genom..

[52] Uncu A.O., Uncu A.T. (2020). High-throughput simple sequence repeat (SSR) mining saturates the carrot (*Daucus carota* L.) genome with chromosome-anchored markers. Biotechnol. Biotechnol. Equip..

[53] Zhao H., Wang W., Yang Y., Wang Z., Sun J., Yuan K., Rabbi S.H.A., Khanam M., Kabir M.S., Seraj Z.I. (2023). A high-quality chromosome-level wild rice genome of *Oryza coarctata*. Sci. Data.

[54] Zhao M., Shu G., Hu Y., Cao G., Wang Y. (2023). Pattern and variation in simple sequence repeat (SSR) at different genomic regions and its implications to maize evolution and breeding. BMC Genom..

[55] Gaikwad A.B., Kumari R., Yadav S., Rangan P., Bhat K. (2023). Small cardamom genome: Development and utilization of microsatellite markers from a draft genome sequence of *Elettaria cardamomum* Maton. Front. Plant Sci..

[56] Kim K.-R., Yu J.-N., Hong J.M., Kim S.-Y., Park S.Y. (2023). Genome assembly and microsatellite marker development using Illumina and PacBio Sequencing in the *Carex pumila* (Cyperaceae) from Korea. Genes.

[57] Caro R.E.S., Cagayan J., Gardoce R.R., Manohar A.N.C., Canama-Salinas A.O., Rivera R.L., Lantican D.V., Galvez H.F., Reaño C.E. (2022). Mining and validation of novel simple sequence repeat (SSR) markers derived from coconut (*Cocos nucifera* L.) genome assembly. J. Genet. Eng. Biotechnol..

[58] Bhattarai G., Shi A., Kandel D.R., Solís-Gracia N., Da Silva J.A., Avila C.A. (2021). Genome-wide simple sequence repeats (SSR) markers discovered from whole-genome sequence comparisons of multiple spinach accessions. Sci. Rep..

[59] Sari D., Sari H., Ikten C., Toker C. (2023). Genome-wide discovery of di-nucleotide SSR markers based on whole genome re-sequencing data of *Cicer arietinum* L. and *Cicer reticulatum* Ladiz. Sci. Rep..

[60] Sayers E.W., Cavanaugh M., Clark K., Pruitt K.D., Sherry S.T., Yankie L., Karsch-Mizrachi I. (2023). GenBank 2023 update. Nucleic Acids Res..

[61] Ewing B., Green P. (1998). Base-calling of automated sequencer traces using phred. II. Error probabilities. Genome Res..

[62] Green P. (1999). Documentation for Phrap and Cross_Match. http://bozeman.mbt.washington.edu/phrap.docs/phrap.html.

[63] Pearson W.R., Lipman D.J. (1988). Improved tools for biological sequence comparison. Proc. Natl. Acad. Sci. USA.

[64] Chen Y., Ye W., Zhang Y., Xu Y. (2015). High speed BLASTN: An accelerated MegaBLAST search tool. Nucleic Acids Res..

[65] Seqclean. https://sourceforge.net/projects/seqclean/.

[66] Hancock J.M., Armstrong J.S. (1994). SIMPLE34: An improved and enhanced implementation for VAX and Sun computers of the SIMPLE algorithm for analysis of clustered repetitive motifs in nucleotide sequences. Bioinformatics.

[67] Morgulis A., Gertz E.M., Schäffer A.A., Agarwala R. (2006). A fast and symmetric DUST implementation to mask low-complexity DNA sequences. J. Comput. Biol..

[68] Bolger A.M., Lohse M., Usadel B. (2014). Trimmomatic: A flexible trimmer for Illumina sequence data. Bioinformatics.

[69] Martin M. (2011). Cutadapt removes adapter sequences from high-throughput sequencing reads. EMBnet J..

[70] Andrews S., Krueger F., Segonds-Pichon A., Biggins L., Krueger C., Wingett S. (2010). FastQC. A Quality Control Tool for High Throughput Sequence Data.

[71] Chen S., Huang T., Zhou Y., Han Y., Xu M., Gu J. (2017). AfterQC: Automatic filtering, trimming, error removing and quality control for fastq data. BMC Bioinform..

[72] Chen S., Zhou Y., Chen Y., Gu J. (2018). fastp: An ultra-fast all-in-one FASTQ preprocessor. Bioinformatics.

[73] Ptitsyn A., Hide W. (2005). CLU: A new algorithm for EST clustering. BMC Bioinform..

[74] Lee Y., Tsai J., Sunkara S., Karamycheva S., Pertea G., Sultana R., Antonescu V., Chan A., Cheung F., Quackenbush J. (2005). The TIGR Gene Indices: Clustering and assembling EST and known genes and integration with eukaryotic genomes. Nucleic Acids Res..

[75] Christoffels A., Gelder A.v., Greyling G., Miller R., Hide T., Hide W. (2001). STACK: Sequence tag alignment and consensus knowledgebase. Nucleic Acids Res..

[76] Chou A., Burke J. (1999). CRAWview: For viewing splicing variation, gene families, and polymorphism in clusters of ESTs and full-length sequences. Bioinformatics.

[77] Huang X., Madan A. (1999). CAP3: A DNA sequence assembly program. Genome Res..

[78] Pertea G., Huang X., Liang F., Antonescu V., Sultana R., Karamycheva S., Lee Y., White J., Cheung F., Parvizi B. (2003). TIGR Gene Indices clustering tools (TGICL): A software system for fast clustering of large EST datasets. Bioinformatics.

[79] Kim S., Lee J. (2006). BAG: A graph theoretic sequence clustering algorithm. Int. J. Data Min. Bioinform..

[80] Merkel A., Gemmell N. (2008). Detecting short tandem repeats from genome data: Opening the software black box. Brief. Bioinform..

[81] Merkel A., Gemmell N.J., Merkel A., Gemmell N.J. (2008). Detecting microsatellites in genome data: Variance in definitions and bioinformatic approaches cause systematic bias. Evol. Bioinform..

[82] Lim K.G., Kwoh C.K., Hsu L.Y., Wirawan A. (2013). Review of tandem repeat search tools: A systematic approach to evaluating algorithmic performance. Brief. Bioinform..

[83] Bergman C.M., Quesneville H. (2007). Discovering and detecting transposable elements in genome sequences. Brief. Bioinform..

[84] Saha S., Bridges S., Magbanua Z.V., Peterson D.G. (2008). Computational approaches and tools used in identification of dispersed repetitive DNA sequences. Trop. Plant Biol..

[85] Lerat E. (2010). Identifying repeats and transposable elements in sequenced genomes: How to find your way through the dense forest of programs. Heredity.

[86] Bao W., Kojima K.K., Kohany O. (2015). Repbase Update, a database of repetitive elements in eukaryotic genomes. Mob. DNA.

[87] Gelfand Y., Rodriguez A., Benson G. (2007). TRDB—The tandem repeats database. Nucleic Acids Res..

[88] Bao Z., Eddy S.R. (2002). Automated de novo identification of repeat sequence families in sequenced genomes. Genome Res..

[89] Price A.L., Jones N.C., Pevzner P.A. (2005). De novo identification of repeat families in large genomes. Bioinformatics.

[90] Koch P., Platzer M., Downie B.R. (2014). RepARK—De novo creation of repeat libraries from whole-genome NGS reads. Nucleic Acids Res..

[91] Stein L.D., Bao Z., Blasiar D., Blumenthal T., Brent M.R., Chen N., Chinwalla A., Clarke L., Clee C., Coghlan A. (2003). The genome sequence of *Caenorhabditis briggsae*: A platform for comparative genomics. PLoS Biol..

[92] Altschul S.F., Gish W., Miller W., Myers E.W., Lipman D.J. (1990). Basic local alignment search tool. J. Mol. Biol..

[93] Bennett M., Leitch I. (2005). Plant genome size research: A field in focus. Ann. Bot..

[94] Kurtz S., Narechania A., Stein J.C., Ware D. (2008). A new method to compute K-mer frequencies and its application to annotate large repetitive plant genomes. BMC Genom..

[95] Ilie L., Ilie S. (2007). Multiple spaced seeds for homology search. Bioinformatics.

[96] Mak D., Gelfand Y., Benson G. (2006). Indel seeds for homology search. Bioinformatics.

[97] Whiteford N., Haslam N., Weber G., Prugel-Bennett A., Essex J., Neylon C. (2008). Visualising the repeat structure of genomic sequences. Complex Syst..

[98] Yoshida T., Obata N., Oosawa K. (2000). Color-coding reveals tandem repeats in the *Escherichia coli* genome. J. Mol. Biol..

[99] Du L., Zhou H., Yan H. (2007). OMWSA: Detection of DNA repeats using moving window spectral analysis. Bioinformatics.

[100] Sharma D., Issac B., Raghava G., Ramaswamy R. (2004). Spectral Repeat Finder (SRF): Identification of repetitive sequences using Fourier transformation. Bioinformatics.

[101] Hauth A.M., Joseph D.A. (2002). Beyond tandem repeats: Complex pattern structures and distant regions of similarity. Bioinformatics.

[102] Kurtz S., Choudhuri J.V., Ohlebusch E., Schleiermacher C., Stoye J., Giegerich R. (2001). REPuter: The manifold applications of repeat analysis on a genomic scale. Nucleic Acids Res..

[103] Abajian C. (1994). Sputnik: DNA Microsatellite Repeat Search Utility.

[104] La Rota M., Kantety R.V., Yu J.-K., Sorrells M.E. (2005). Nonrandom distribution and frequencies of genomic and EST-derived microsatellite markers in rice, wheat, and barley. BMC Genom..

[105] Smit A., Hubley R., Green P. (2004). RepeatMasker Open-3.0. http://www.repeatmasker.org.

[106] Bedell J.A., Korf I., Gish W. (2000). MaskerAid: A performance enhancement to RepeatMasker. Bioinformatics.

[107] Tarailo-Graovac M., Chen N. (2009). Using Repeat Masker to identify repetitive elements in genomic sequences. Curr. Protoc. Bioinform..

[108] Benson G. (1999). Tandem repeats finder: A program to analyze DNA sequences. Nucleic Acids Res..

[109] Castelo A.T., Martins W., Gao G.R. (2002). TROLL—Tandem repeat occurrence locator. Bioinformatics.

[110] Duran C., Appleby N., Edwards D., Batley J. (2009). Molecular genetic markers: Discovery, applications, data storage and visualisation. Curr. Bioinform..

[111] Thiel T., Michalek W., Varshney R., Graner A. (2003). Exploiting EST databases for the development and characterization of gene-derived SSR-markers in barley (*Hordeum vulgare* L.). Theor. Appl. Genet..

[112] Beier S., Thiel T., Münch T., Scholz U., Mascher M. (2017). MISA-web: A web server for microsatellite prediction. Bioinformatics.

[113] Bizzaro J.W., Marx K.A. (2003). Poly: A quantitative analysis tool for simple sequence repeat (SSR) tracts in DNA. BMC Bioinform..

[114] Parisi V., De Fonzo V., Aluffi-Pentini F. (2003). STRING: Finding tandem repeats in DNA sequences. Bioinformatics.

[115] Bilgen M., Karaca M., Onus A.N., Ince A.G. (2004). A software program combining sequence motif searches with keywords for finding repeats containing DNA sequences. Bioinformatics.

[116] Karaca M., Bilgen M., Onus A.N., Ince A.G., Elmasulu S.Y. (2005). Exact tandem repeats analyzer (E-TRA): A new program for DNA sequence mining. J. Genet..

[117] Wexler Y., Yakhini Z., Kashi Y., Geiger D. (2005). Finding approximate tandem repeats in genomic sequences. J. Comput. Biol..

[118] Anwar T., Khan A.U. (2006). SSRscanner: A program for reporting distribution and exact location of simple sequence repeats. Bioinformation.

[119] Boeva V., Regnier M., Papatsenko D., Makeev V. (2006). Short fuzzy tandem repeats in genomic sequences, identification, and possible role in regulation of gene expression. Bioinformatics.

[120] Kofler R., Schlötterer C., Lelley T. (2007). SciRoKo: A new tool for whole genome microsatellite search and investigation. Bioinformatics.

[121] Fonzo V.D., Aluffi-Pentini F., Parisi V. (2008). JSTRING: A novel Java tandem repeats searcher in genomic sequences with an interactive graphic output. Open Appl. Inform. J..

[122] Banerjee N., Chidambarathanu N., Michael D., Balakrishnan N., Sekar K. (2008). An algorithm to find all identical internal sequence repeats. Curr. Sci..

[123] Senthilkumar R., Sabarinathan R., Hameed B.S., Banerjee N., Chidambarathanu N., Karthik R., Sekar K. (2010). FAIR: A server for internal sequence repeats. Bioinformation.

[124] Pai T.-W., Chen C.-M., Hsiao M.-C., Cheng R., Tzou W.-S., Hu C.-H. (2009). An online conserved SSR discovery through cross-species comparison. Adv. Appl. Bioinform. Chem..

[125] Jorda J., Kajava A.V. (2009). T-REKS: Identification of Tandem REpeats in sequences with a K-meanS based algorithm. Bioinformatics.

[126] Chen M., Tan Z., Zeng G. (2011). MfSAT: Detect simple sequence repeats in viral genomes. Bioinformation.

[127] Wang X., Lu P., Luo Z. (2013). GMATo: A novel tool for the identification and analysis of microsatellites in large genomes. Bioinformation.

[128] Lopes R.d.S., Moraes W.J.L., Rodrigues T.d.S., Bartholomeu D.C. (2015). ProGeRF: Proteome and genome repeat finder utilizing a fast parallel hash function. BioMed Res. Int..

[129] Weiner P. Linear pattern matching algorithms. Proceedings of the 14th Annual Symposium on Switching and Automata Theory (Swat 1973).

[130] Pickett B.D., Karlinsey S., Penrod C., Cormier M.J., Ebbert M.T., Shiozawa D.K., Whipple C., Ridge P.G. (2016). SA-SSR: A suffix array-based algorithm for exhaustive and efficient SSR discovery in large genetic sequences. Bioinformatics.

[131] Pickett B.D., Miller J.B., Ridge P.G. (2017). Kmer-SSR: A fast and exhaustive SSR search algorithm. Bioinformatics.

[132] Avvaru A.K., Sowpati D.T., Mishra R.K. (2018). PERF: An exhaustive algorithm for ultra-fast and efficient identification of microsatellites from large DNA sequences. Bioinformatics.

[133] Gou X., Ma J., Liu Y. (2020). SSRMMD: A rapid and accurate algorithm for mining SSR feature loci and candidate polymorphic SSRs based on assembled sequences. Front. Genet..

[134] Alves S.I.A., Ferreira V.B.C., Dantas C.W.D., Silva A.L.d.C.d., Ramos R.T.J. (2023). EasySSR: A user-friendly web application with full command-line features for large-scale batch microsatellite mining and samples comparison. Front. Genet..

[135] Volfovsky N., Haas B.J., Salzberg S.L. (2001). A clustering method for repeat analysis in DNA sequences. Genome Biol..

[136] Kolpakov R., Bana G., Kucherov G. (2003). mreps: Efficient and flexible detection of tandem repeats in DNA. Nucleic Acids Res..

[137] Warburton P.E., Giordano J., Cheung F., Gelfand Y., Benson G. (2004). Inverted repeat structure of the human genome: The X-chromosome contains a preponderance of large, highly homologous inverted repeats that contain testes genes. Genome Res..

[138] Delgrange O., Rivals E. (2004). STAR: An algorithm to search for tandem approximate repeats. Bioinformatics.

[139] Krishnan A., Tang F. (2004). Exhaustive whole-genome tandem repeats search. Bioinformatics.

[140] Kumpatla S.P., Mukhopadhyay S. (2005). Mining and survey of simple sequence repeats in expressed sequence tags of dicotyledonous species. Genome.

[141] Thurston M., Field D. (2006). Msatfinder: Detection and Characterisation of Microsatellites.

[142] de Ridder C., Kourie D.G., Watson B.W. FireµSat: An algorithm to detect microsatellites in DNA. Proceedings of the Prague Stringology Conference.

[143] de Ridder C., Kourie D.G., Watson B.W., Fourie T., Reyneke P. (2013). Fine-tuning the search for microsatellites. J. Discret. Algorithms.

[144] Mayer C. (2008). Phobos, a tandem repeat search tool for complete genomes. Version.

[145] Mudunuri S.B., Nagarajaram H.A. (2007). IMEx: Imperfect microsatellite extractor. Bioinformatics.

[146] Faircloth B.C. (2008). MSATCOMMANDER: Detection of microsatellite repeat arrays and automated, locus-specific primer design. Mol. Ecol. Resour..

[147] Otto T.D., Gomes L.H., Alves-Ferreira M., de Miranda A.B., Degrave W.M. (2008). ReRep: Computational detection of repetitive sequences in genome survey sequences (GSS). BMC Bioinform..

[148] da Maia L.C., Palmieri D.A., de Souza V.Q., Kopp M.M., de Carvalho F.I.F., Costa de Oliveira A. (2008). SSR locator: Tool for simple sequence repeat discovery integrated with primer design and PCR simulation. Int. J. Plant Genom..

[149] Abraham A.-L., Rocha E.P., Pothier J. (2008). Swelfe: A detector of internal repeats in sequences and structures. Bioinformatics.

[150] Pellegrini M., Renda M., Vecchio A. (2010). TRStalker: An efficient heuristic for finding fuzzy tandem repeats. Bioinformatics.

[151] Catanese H.N., Brayton K.A., Gebremedhin A.H. (2016). RepeatAnalyzer: A tool for analysing and managing short-sequence repeat data. BMC Genom..

[152] Untergasser A., Cutcutache I., Koressaar T., Ye J., Faircloth B.C., Remm M., Rozen S.G. (2012). Primer3—new capabilities and interfaces. Nucleic Acids Res..

[153] Rychlik W. (2007). OLIGO 7 primer analysis software. PCR primer design. Methods Mol. Biol..

[154] You F.M., Huo N., Gu Y.Q., Luo M.-c., Ma Y., Hane D., Lazo G.R., Dvorak J., Anderson O.D. (2008). BatchPrimer3: A high throughput web application for PCR and sequencing primer design. BMC Bioinform..

[155] Ye J., Coulouris G., Zaretskaya I., Cutcutache I., Rozen S., Madden T.L. (2012). Primer-BLAST: A tool to design target-specific primers for polymerase chain reaction. BMC Bioinform..

[156] Kalendar R., Lee D., Schulman A.H. (2009). FastPCR software for PCR primer and probe design and repeat search. Genes Genomes Genom..

[157] Kalendar R., Lee D., Schulman A.H. (2014). FastPCR software for PCR, in silico PCR, and oligonucleotide assembly and analysis. DNA Cloning Assem. Methods.

[158] Sreenu V.B., Alevoor V., Nagaraju J., Nagarajaram H.A. (2003). MICdb: Database of prokaryotic microsatellites. Nucleic Acids Res..

[159] Sreenu V.B., Ranjitkumar G., Swaminathan S., Priya S., Bose B., Pavan M.N., Thanu G., Nagaraju J., Nagarajaram H.A. (2003). MICAS: A fully automated web server for microsatellite extraction and analysis from prokaryote and viral genomic sequences. Appl. Bioinform..

[160] Robinson A.J., Love C.G., Batley J., Barker G., Edwards D. (2004). Simple sequence repeat marker loci discovery using SSR primer. Bioinformatics.

[161] Jewell E., Robinson A., Savage D., Erwin T., Love C.G., Lim G.A., Li X., Batley J., Spangenberg G.C., Edwards D. (2006). SSRPrimer and SSR taxonomy tree: Biome SSR discovery. Nucleic Acids Res..

[162] Fukuoka H., Nunome T., Minamiyama Y., Kono I., Namiki N., Kojima A. (2005). Read2Marker: A data processing tool for microsatellite marker development from a large data set. Biotechniques.

[163] Tang J., Baldwin S.J., Jacobs J.M., van der Linden C.G., Voorrips R.E., Leunissen J.A., van Eck H., Vosman B. (2008). Large-scale identification of polymorphic microsatellites using an in silico approach. BMC Bioinform..

[164] Martins W.S., Lucas D.C.S., de Souza Neves K.F., Bertioli D.J. (2009). WebSat-A web software for microsatellite marker development. Bioinformation.

[165] Sarmah R., Sahu J., Dehury B., Sarma K., Sahoo S., Sahu M., Barooah M., Sen P., Modi M.K. (2012). ESMP: A high-throughput computational pipeline for mining SSR markers from ESTs. Bioinformation.

[166] Churbanov A., Ryan R., Hasan N., Bailey D., Chen H., Milligan B., Houde P. (2012). HighSSR: High-throughput SSR characterization and locus development from next-gen sequencing data. Bioinformatics.

[167] Meglécz E., Costedoat C., Dubut V., Gilles A., Malausa T., Pech N., Martin J.-F. (2010). QDD: A user-friendly program to select microsatellite markers and design primers from large sequencing projects. Bioinformatics.

[168] Meglécz E., Pech N., Gilles A., Dubut V., Hingamp P., Trilles A., Grenier R., Martin J.F. (2014). QDD version 3.1: A user-friendly computer program for microsatellite selection and primer design revisited: Experimental validation of variables determining genotyping success rate. Mol. Ecol. Resour..

[169] Wang X., Wang L. (2016). GMATA: An integrated software package for genome-scale SSR mining, marker development and viewing. Front. Plant Sci..

[170] Ponyared P., Ponsawat J., Tongsima S., Seresangtakul P., Akkasaeng C., Tantisuwichwong N. (2016). ESAP plus: A web-based server for EST-SSR marker development. BMC Genom..

[171] Xia E.-H., Yao Q.-Y., Zhang H.-B., Jiang J.-J., Zhang L.-P., Gao L.-Z. (2016). CandiSSR: An efficient pipeline used for identifying candidate polymorphic SSRs based on multiple assembled sequences. Front. Plant Sci..

[172] Metz S., Cabrera J.M., Rueda E., Giri F., Amavet P. (2016). FullSSR: Microsatellite finder and primer designer. Adv. Bioinform..

[173] Pandey M., Kumar R., Srivastava P., Agarwal S., Srivastava S., Nagpure N.S., Jena J.K., Kushwaha B. (2018). WGSSAT: A high-throughput computational pipeline for mining and annotation of SSR markers from whole genomes. J. Hered..

[174] Guang X.-M., Xia J.-Q., Lin J.-Q., Yu J., Wan Q.-H., Fang S.-G. (2019). IDSSR: An efficient pipeline for identifying polymorphic microsatellites from a single genome sequence. Int. J. Mol. Sci..

[175] Alves F., Martins F.M., Areias M., Muñoz-Mérida A. (2022). Automating microsatellite screening and primer design from multi-individual libraries using Micro-Primers. Sci. Rep..

[176] Mokhtar M.M., Alsamman A.M., El Allali A. (2023). MegaSSR: A web server for large scale microsatellite identification, classification, and marker development. Front. Plant Sci..

[177] Leclercq S., Rivals E., Jarne P. (2007). Detecting microsatellites within genomes: Significant variation among algorithms. BMC Bioinform..

[178] Chen C., Chen C., Shih T., Pai T., Hu C., Tzou W. (2010). Efficient algorithms for identifying orthologous simple sequence repeats of disease genes. J. Syst. Sci. Complex..

[179] Mathur M. (2020). A comparative study of various SSRs identification tools using *Aspergillus Fumigatus* chromosome sequences. J. Bioinform. Comp. Genom..

[180] Landau G.M., Schmidt J.P., Sokol D. (2001). An algorithm for approximate tandem repeats. J. Comput. Biol..

[181] Elliott T.A., Heitkam T., Hubley R., Quesneville H., Suh A., Wheeler T.J., TE Hub Consortium (2021). TE Hub: A community-oriented space for sharing and connecting tools, data, resources, and methods for transposable element annotation. Mob DNA.

[182] Aishwarya V., Grover A., Sharma P.C. (2007). EuMicroSat db: A database for microsatellites in the sequenced genomes of eukaryotes. BMC Genom..

[183] Aishwarya V., Sharma P.C. (2007). UgMicroSat db: Database for mining microsatellites from unigenes. Nucleic Acids Res..

[184] Avvaru A.K., Saxena S., Sowpati D.T., Mishra R.K. (2017). MSDB: A comprehensive database of simple sequence repeats. Genome Biol. Evol..

[185] Avvaru A.K., Sharma D., Verma A., Mishra R.K., Sowpati D.T. (2020). MSDB: A comprehensive, annotated database of microsatellites. Nucleic Acids Res..

[186] Kumar P., Chaitanya P.S., Nagarajaram H.A. (2010). PSSRdb: A relational database of polymorphic simple sequence repeats extracted from prokaryotic genomes. Nucleic Acids Res..

[187] Mokhtar M.M., Atia M.A.M. (2019). SSRome: An integrated database and pipelines for exploring microsatellites in all organisms. Nucleic Acids Res..

[188] Subramanian S., Madgula V.M., George R., Mishra R.K., Pandit M.W., Kumar C.S., Singh L. (2002). MRD: A microsatellite repeats database for prokaryotic and eukaryotic genomes. Genome Biol..

[189] Boby T., Patch A.-M., Aves S. (2005). TRbase: A database relating tandem repeats to disease genes for the human genome. Bioinformatics.

[190] Chang Y.-H., Su W.-H., Lee T.-C., Sun H.-F.S., Chen C.-H., Pan W.-H., Tsai S.-F., Jou Y.-S. (2005). TPMD: A database and resources of microsatellite marker genotyped in Taiwanese populations. Nucleic Acids Res..

[191] Missirlis P.I., Mead C.-L.R., Butland S.L., Ouellette B.F., Devon R.S., Leavitt B.R., Holt R.A. (2005). Satellog: A database for the identification and prioritization of satellite repeats in disease association studies. BMC Bioinform..

[192] Subramanian S., Madgula V.M., George R., Kumar S., Pandit M.W., Singh L. (2003). SSRD: Simple sequence repeats database of the human genome. Comp. Funct. Genom..

[193] Sakai T., Miura I., Yamada-Ishibashi S., Wakita Y., Kohara Y., Yamazaki Y., Inoue T., Kominami R., Moriwaki K., Shiroishi T. (2004). Update of mouse microsatellite database of Japan (MMDBJ). Exp. Anim..

[194] Archak S., Meduri E., Kumar P.S., Nagaraju J. (2007). InSatDb: A microsatellite database of fully sequenced insect genomes. Nucleic Acids Res..

[195] Prasad M., Muthulakshmi M., Arunkumar K., Madhu M., Sreenu V.B., Pavithra V., Bose B., Nagarajaram H.A., Mita K., Shimada T. (2005). SilkSatDb: A microsatellite database of the silkworm, Bombyx mori. Nucleic Acids Res..

[196] Karaoglu H., Lee C.M.Y., Meyer W. (2005). Survey of simple sequence repeats in completed fungal genomes. Mol. Biol. Evol..

[197] Mudunuri S., Appa Rao A., Pallamsetty S., Mishra P., Nagarajaram H. (2009). VMD: Viral Microsatellite Database-A Comprehensive Resource for all Viral Microsatellites. J. Comput. Sci. Syst. Biol..

[198] Arora V., Kapoor N., Fatma S., Jaiswal S., Iquebal M.A., Rai A., Kumar D. (2018). BanSatDB, a whole-genome-based database of putative and experimentally validated microsatellite markers of three Musa species. Crop J..

[199] Arumugam V., Riju A., Arunachalam V. Mining of expressed sequence tag (EST) libraries and core nucleotide sequences for simple sequence repeats (SSR) in papaya. Proceedings of the II International Symposium on Papaya, Madurai.

[200] Babu B.K., Rani K.M., Sahu S., Mathur R., Kumar P.N., Ravichandran G., Anitha P., Bhagya H. (2019). Development and validation of whole genome-wide and genic microsatellite markers in oil palm (Elaeis guineensis Jacq.): First microsatellite database (OpSatdb). Sci. Rep..

[201] Blenda A., Scheffler J., Scheffler B., Palmer M., Lacape J.-M., Yu J.Z., Jesudurai C., Jung S., Muthukumar S., Yellambalase P. (2006). CMD: A cotton microsatellite database resource for Gossypium genomics. BMC Genom..

[202] Channdrasekar A., Rijju A., Sathyanath N.V., Santhosh E. (2009). SpicEST-An Annotated database on Expressed Sequence tags of spices. Genes Genomes Genom..

[203] Duhan N., Meshram M., Loaiza C.D., Kaundal R. (2020). citSATdb: Genome-wide simple sequence repeat (SSR) marker database of Citrus species for germplasm characterization and crop improvement. Genes.

[204] Jayashree B., Punna R., Prasad P., Bantte K., Hash C.T., Chandra S., Hoisington D.A., Varshney R.K. (2006). A database of simple sequence repeats from cereal and legume expressed sequence tags mined in silico: Survey and evaluation. Silico Biol..

[205] Mueller L.A., Solow T.H., Taylor N., Skwarecki B., Buels R., Binns J., Lin C., Wright M.H., Ahrens R., Wang Y. (2005). The SOL Genomics Network. A comparative resource for Solanaceae biology and beyond. Plant Physiol..

[206] Portis E., Portis F., Valente L., Moglia A., Barchi L., Lanteri S., Acquadro A. (2016). A genome-wide survey of the microsatellite content of the globe artichoke genome and the development of a web-based database. PLoS ONE.

[207] Purru S., Sahu S., Rai S., Rao A., Bhat K. (2018). GinMicrosatDb: A genome-wide microsatellite markers database for sesame (*Sesamum indicum* L.). Physiol. Mol. Biol. Plants.

[208] Shirasawa K., Asamizu E., Fukuoka H., Ohyama A., Sato S., Nakamura Y., Tabata S., Sasamoto S., Wada T., Kishida Y. (2010). An interspecific linkage map of SSR and intronic polymorphism markers in tomato. Theor. Appl. Genet..

[209] Song X., Yang Q., Bai Y., Gong K., Wu T., Yu T., Pei Q., Duan W., Huang Z., Wang Z. (2021). Comprehensive analysis of SSRs and database construction using all complete gene-coding sequences in major horticultural and representative plants. Hortic. Res..

[210] Youens-Clark K., Buckler E., Casstevens T., Chen C., DeClerck G., Derwent P., Dharmawardhana P., Jaiswal P., Kersey P., Karthikeyan A. (2010). Gramene database in 2010: Updates and extensions. Nucleic Acids Res..

[211] Yu J., Dossa K., Wang L., Zhang Y., Wei X., Liao B., Zhang X. (2017). PMDBase: A database for studying microsatellite DNA and marker development in plants. Nucleic Acids Res..

[212] Du L., Liu Q., Zhao K., Tang J., Zhang X., Yue B., Fan Z. (2020). PSMD: An extensive database for pan-species microsatellite investigation and marker development. Mol. Ecol. Resour..

