# Supplementary material for: Streamlining of Simple Sequence Repeat Data Mining Methodologies and Pipelines for Crop Scanning
Source: Plants (Basel). 2024 Sep 19;13(18):2619. doi: 10.3390/plants13182619 (PMC11435353; doi:10.3390/plants13182619)
Supplement: Supplementary file 1 [file plants-13-02619-s001.zip › plants-3097054-supplementary.pdf]

Supplementary Table S1: Features of Primer Designing Software

Software	Availability	System requirements	Special features	References/URL
Primer 3	Free	Online version	It is the widely used primer designing software, easy and simple to use with flexibility in adjusting the parameters	[152] https://primer3.org/
Primer Premier	On purchase	Mac	First software to fully integrate multiple sequence alignment with primer design It allows complete control over primer design to the user. Easy and simple to design cross species and allele specific primers	-
Oligo	Purchase	Platform independent Java script	First version appeared in 1989. The latest version is Oligo 7. The special features include batch processing of sequence files covering multiple DNA regions, selection of multiplex primers and nested primer sets in just one search, and graphical display of oligos	[153] http://www.oligo.net/
ORFprimer	Free	Platform independent Java script	Simple in handling with a graphical user interface and can compute thousands of primers for up to thousands of target sequences	http://www.proteinstruktu rfabrik.de/ORFprimer/
Batch Primer3	Free	Online version	Adopted based on primer3 for high throughput primer designing	[154] http://wheat.pw.usda.gov/ demos/BatchPrimer3/.
Primer Blast	Free	Online version	It is a robust primer designing tool for designing target specific primers and offers flexible options to adjust specificity thresholds	[155] https://www.ncbi.nlm.nih. gov/tools/primer-blast/
Primer quest	Free	Online version	It has 3 versions *viz.*, basic, standard and advanced versions for primer designing. In basic version, only default settings can be applied, however standard and advanced versions permit several user defined options	http://eu.idtdna.com/Scito ols/Applications/Primerqu est/Advanced.aspx
Net primer	Free	Online version	It combines the latest primer analysis algorithms with a web-based interface. Primers are analyzed for melting temperature and secondary structures. Quantitation of primers is made easy by calculating primer molecular weight and optical activity. To facilitate the selection of an optimal primer, each primer is given a rating based on the stability of its secondary structures. A comprehensive analysis report can be printed for individual primers or primer pairs.	http://www.premierbiosof t.com/netprimer/index.ht ml
NoePrimer	Free trial versions available	Windows, Linux, Macintosh	Easy and simple for high throughput primer search and analysis. It supports multiple PCR primer analysis. It provides a graphical representation of the primers designed which facilitates visualization of secondary structures and false priming sites. It also tracks and saves primers to the primer library.	http://www.noegen.com/e n/index.php
FastPCR	Free	Windows	It is an integrated tool that allows primer designing, sequence editing, alignment, database analysis and also repeat searching. It has the ability to handle long sequences	[156, 157] https://primerdigital.com/f astpcr.html

Supplementary Table S2: Features of web-based resources specifically created for accessing information on microsatellites in various organisms

Database	URL	Features	Datamining tool used	Reference
Microsatellite repeat database (MRD)	http://insilico.ehu.es/microsatellites/info.html	Perfect and imperfect microsatellites in sequenced prokaryotes and eukaryotes	Microsatellite repeat finder	[188]
Simple sequence repeat database	http://www.ccmb.res.in/ssr<br>http://www.ingenovis.com/ssr.	Perfect simple sequence repeats in human genome	K mer based search	[192]
MICdb -Microsatellites database<br>Microsatellite analysis Server 3.0 (MICAS)	(http://www.cdfd.org.in/micas)<br><br>http://www.mcr.org.in/micas/	Perfect microsatellites from fully sequenced prokaryote and viral genomes (>83 genomes)<br>Online repository of prokaryote and viral genome	WSSRF<br><br>Imex	[158]<br>[159]
MMDBJ<br>Mouse Microsatellite Database of Japan	www.shigen.nig.ac.jp/mouse/mmdbj/	Microsatellites from four *Mus musculus* subspecies	-	[193]
TRbase	http://trbase.ex.ac.<br>Uk	Perfect and imperfect microsatellite collection of human and their relation to disease genes	TRF	[189]
Taiwan Polymorphic Microsatellite Database	http://tpmd.nhri.org.tw/	Human microsatellites (Taiwanese populations)	-	[190]
Database of Molecular Mycology Research Lab	www.mmrl.med.usyd.edu.au/ssr.html	Perfect microsatellite repeats from nine fungal genomes	Python based algorithm	[196]
Satellog	http://satellog.bcgsc.ca	Perfect repeats of 1-16 motifs from the human genome	TRF	[191]
SGN<br>Sol Genomics Network	http://solgenomics.net/	Mono to hexa nucleotide perfect repeats extracted from solanaceaous crops	Analytical tools developed by Genomics Edge Technologies, Inc; St. Louis, Missouri	[205]
Silk satDb	http://www.cdfd.org.in/SILKSAT/index.php?f=silkhome	Microsatellites extracted from WGS and EST sequences of *Bombyx moori*	SSRF	[195]
CMD<br>Cotton marker Database	http://www.cottonssr.org/	Annotated Collection of 5484microsatellites from nine cotton microsatellite projects	SSRIT	[201]
SSR database	http://www.intranet.icrisat.org/gt1/SSR/S	Microsatellites form ESTS of Sorghum, Glycine	SSRIT	[204]

	SRdatabase.html	max, Lotus japonica, Oryza sativa, Zea mays, Medicago truncatula		
EuMicroSat*db*<br>**Eu**karyotic **MicroSat**ellite *data*b*a*se	http://www.veenuash.info/<br>http://ipu.ac.in/usbt/EuMicroSatdb.htm	Microsatellites in the fully sequenced genomes of eukaryotes	MISA	[182]
InSatDb	http://210.212.212.8/PHP/INSATDB/home.php	Microsatellites from five insect genomes	TRF	[194]
TRDB<br>Tandem repeat database	https://tandem.bu.edu/cgi-bin/trdb/trdb.exe	Microsatellites from archival copies of 22 genomes available in public databases	TRF	[87]
UgMicroSatdb<br>Unigene MicroSatellite database	http://ipu.ac.in/usbt/UgMicroSatdb.htm.	Microsatellites from unigenes of more than 80 eukaryotic genomes	MISA	[183]
Papaya SSR DB	http://riju.byethost31.com/papaya/index.html	Perfect and compound repeats extracted from papaya ESTs	MISA and eTRA	[199]
VMD-Viral microsatellite database	http://www.mcr.org.in/vmd/index.php	Perfect and imperfect microsatellites from 3000 viral genomes	Imex	[197]
SpicEST database	http://220.227.138.213/spiceest/index.html<br>http://www.spices.res.in/spiceest	Microsatellite extracted from the ESTs of ginger and turmeric	MISA, ETRA, SSR PRIMER, SSRIT, WEB TROLL	[202]
Tomato marker database	http://marker.kazusa.or.jp/Tomato/	Collection of EST and genomic SSRs of tomato	Find patterns	[208]
PSSRdb<br>Polymorphic simple sequence repeat database	http://pssrdb.cdfd.org.in/intro.html	Perfect repeats of 85 prokaryotic species	SSRF	[186]
Gramene database	http://www.gramene.org/markers/	Microsatellite marker information in rice	SSRIT	[210]
Microsat2006	www.microsatellites.org/db_search.php	Human microsatellites		-
Small genomes microsatellite database (SGMD)	http://www.genomics.ceh.ac.uk/cgi-bin/sgmd/index.cgi	Imperfect Microsatellites from bacterial, organellar, plasmid and viral genomes	Msat finder	-
Cynara cardunculus microsatellite database (CyMSatDB)	http://www.artichokegenome.unito.it/cymsatdb/	Perfect, compound and imperfect repeat SSRs from *Cynara cardunculus*	SciRoKo	[206]
MSDB	http://tdb.ccmb.res.in/msdb	Millions of SSRs from 6893 species including bacteria, fungi, archae, plants and animals	Custom Python script	[184, 185]
PMDBase	http://www.sesame-bioinfo.org/PMDBase	Microsatellites from 110 eukaryotic species	MISA	[211]
BanSatDB	http://webtom.cabgrid.res.in/bansatdb/	Genome wide SSRs from three Musa species	MISA and custom	[198]

			Perl script	
SSRome	http://mggm-lab.easyomics.org	Perfect and compound microsatellites from 6533 organisms	MISA	[187]
GinMicroSatDB	http://backwin.cabgrid.res.in:8080/Gingelly7.	Genome wide perfect and compound SSR from sesame	MISA	[207]
opSatdb	https://ssr.icar.gov.in/index.php	Genic and genome wide SSR from oilpalm	Websat, MISA	[200]
citSATdb	https://bioinfo.usu.edu/citSATdb/	SSR from eight species of Citrus	miSATminer	[203]
PSMD	http://big.cdu.edu.cn/psmd/	Pan-species microsatellites from 18408 organisms		[212]
Plant SSR Database (PSSRD)	http://www.pssrd.info/	Genic SSR from 112 plants	MISA	[209]
